# Molecular Mechanisms of Nemorosone-Induced Ferroptosis in Cancer Cells

**DOI:** 10.3390/cells12050735

**Published:** 2023-02-24

**Authors:** Roberto Fernández-Acosta, Behrouz Hassannia, Jurgen Caroen, Bartosz Wiernicki, Daniel Alvarez-Alminaque, Bruno Verstraeten, Johan Van der Eycken, Peter Vandenabeele, Tom Vanden Berghe, Gilberto L. Pardo-Andreu

**Affiliations:** 1 Department of Pharmacy, Institute of Pharmacy and Food, University of Havana, 222 St. # 2317, La Coronela, La Lisa, Havana 13600, Cuba; 2 Cell Death and Inflammation Unit, VIB Center for Inflammation Research (IRC), 9052 Ghent, Belgium; 3 Department of Biomedical Molecular Biology (DBMB), Ghent University, 9052 Ghent, Belgium; 4 Laboratory of Pathophysiology, Department of Biomedical Sciences, University of Antwerp, 2000 Antwerp, Belgium; 5 Laboratory for Organic and Bio-Organic Synthesis, Department of Organic and Macromolecular Chemistry, Ghent University, 9000 Ghent, Belgium; 6 Center for Research and Biological Evaluations, Institute of Pharmacy and Food, University of Havana, 222 St. # 2317, La Coronela, La Lisa, Havana 13600, Cuba; 7 Methusalem Program, Ghent University, 9052 Ghent, Belgium

**Keywords:** nemorosone, ferroptosis, mitochondrial uncoupling, fibrosarcoma, neuroblastoma

## Abstract

Ferroptosis is an iron-dependent cell death-driven by excessive peroxidation of polyunsaturated fatty acids (PUFAs) of membranes. A growing body of evidence suggests the induction of ferroptosis as a cutting-edge strategy in cancer treatment research. Despite the essential role of mitochondria in cellular metabolism, bioenergetics, and cell death, their function in ferroptosis is still poorly understood. Recently, mitochondria were elucidated as an important component in cysteine-deprivation-induced (CDI) ferroptosis, which provides novel targets in the search for new ferroptosis-inducing compounds (FINs). Here, we identified the natural mitochondrial uncoupler nemorosone as a ferroptosis inducer in cancer cells. Interestingly, nemorosone triggers ferroptosis by a double-edged mechanism. In addition to decreasing the glutathione (GSH) levels by blocking the System xc cystine/glutamate antiporter (SLC7A11), nemorosone increases the intracellular labile Fe^2+^ pool via heme oxygenase-1 (HMOX1) induction. Interestingly, a structural variant of nemorosone (*O*-methylated nemorosone), having lost the capacity to uncouple mitochondrial respiration, does not trigger cell death anymore, suggesting that the mitochondrial bioenergetic disruption via mitochondrial uncoupling is necessary for nemorosone-induced ferroptosis. Our results open novel opportunities for cancer cell killing by mitochondrial uncoupling-induced ferroptosis.

## 1. Introduction

The natural product nemorosone was isolated in 1996 through the extraction of floral resins from *Clusia rosea*, an evergreen tropical plant with several medicinal applications [1]. Its chemical structure was unraveled in 2001 as a type A polyisoprenylated benzophenone [2]. The high interest in nemorosone mainly resides in its cytotoxic anti-cancer activity, as shown in a broad spectrum of different human cancer models such as leukemia, colorectal, pancreatic, hepatic, and breast cancer [3,4,5,6,7,8,9,10,11]. In most cases, apoptosis was reported as its mode of cytotoxicity along with the arrest of cell cycle progression. Recently, its antimetastatic potential through the modulation of molecules related to the epithelial-mesenchymal transition (EMT) was also described [11]. Furthermore, several research works have reported antimutagenic activity without a genotoxic effect, selectivity toward cancer cells, and the capacity to circumvent multidrug-resistance mechanisms [4,5,12,13,14]. Altogether, these previous studies show that nemorosone can be considered as a lead compound for the development of novel antiproliferative drugs for cancer therapy.

On the other hand, we found that nemorosone disrupts the mitochondrial bioenergetic status by acting as a potent protonophoric mitochondrial uncoupler [6,9]. Therefore, despite the well-established capacity of nemorosone to induce apoptosis [4,5], other modes of regulated cell death could be induced depending on the cell type. Since the conceptualization of ferroptosis in 2012, several compounds previously described as inducers of apoptosis, such as cisplatin, sorafenib, and withaferin A, have been found to elicit ferroptosis [15,16,17]. Moreover, the induction of ferroptosis has also been identified in a myriad of natural products [18].

Ferroptosis is a form of regulated necrosis mediated by iron-catalyzed excessive lipid peroxidation [19,20], often referred to as biological rust of lipid membranes [21]. Ferroptosis can be induced by inactivating glutathione peroxidase 4 (GPX4), which detoxifies lipid hydroperoxides. GPX4 can be inactivated through direct targeting and inhibition, by class II and III inducers, or alternatively by indirect mechanisms through the depletion of intracellular GSH, an essential co-factor of GPX4, by class I inducers [22]. Furthermore, ferroptosis can be activated by the increase in the cellular labile iron pool (LIP), which is the intracellular non-protein bound redox-active iron, or iron oxidation mediated by class IV inducers [21].

Recently, Gao et al. showed that mitochondria play a central role in cysteine-deprivation-induced and erastin-induced ferroptosis (Class I FINs), but not in the case of the ferroptosis induced by GPX4 inhibition (Class II FINs). Mechanistically, cysteine deprivation leads to a transient hyperpolarization of the mitochondrial membrane potential and lipid peroxide production [23]. On the other hand, cell death induced by mitochondrial uncoupling is accompanied by depolarization of the mitochondrial membrane potential, reduced ATP levels, increased ROS, and diminished antioxidant defense by decreasing GSH levels [24]. The latter may resemble a similar cellular effect observed in erastin-treated cells. Therefore, we hypothesized and examined whether mitochondrial uncoupling by nemorosone could initiate a ferroptotic response.

Using different cell lines, we found that nemorosone triggers ferroptosis, as detected by lipid peroxidation. We elucidated that nemorosone-induced ferroptosis involves a double-edged mechanism. Nemorosone decreases the glutathione levels by blocking the cystine/glutamate antiporter and induces lipid peroxidation as an early event. At later time points, nemorosone administration results in activation of the KEAP1–NRF2–HMOX1 axis, causing an increase in the intracellular labile Fe^2+^ pool and consequent reactive oxygen species (ROS) production. Methylnemorosone, a structural variant of nemorosone that has lost the capacity to uncouple mitochondrial respiration, does not trigger cell death anymore. The classical mitochondrial uncoupler carbonyl cyanide 3-chlorophenylhydrazone (CCCP) has a similar biological effect as nemorosone, in inducing ferroptosis. In summary, our results demonstrate that nemorosone as well as other mitochondrial uncouplers drive intrinsic ferroptosis. The present findings could open new perspectives for a better insight into ferroptosis initiated by mitochondrial dysfunction and for the development of novel ferroptosis inducers for cancer treatment.

## 2. Materials and Methods

### 2.1. Antibodies and Reagents

The following antibodies were used in this study: β-tubulin (Abcam, Cambridge, UK, ab21058), GPX4 (Abcam, ab41787), HMOX1 (Enzo Life Sciences, Farmingdale, NY, USA, ADI-SPA-896-F), NRF2 (Abcam, ab62352), and KEAP1 (Proteintech, Rosemont, IL, USA, 10503-2-AP). The following chemicals were used: SytoxGreen (Thermo Fisher Scientific, Waltham, MA, USA, S7020): 1.7 μM, BODIPY 581/591 C11 probe (Invitrogen, Waltham, MA, USA, D-3861): 2 μM, SytoxBlue (Thermo Fisher Scientific, S11348): 1.25 μM, DRAQ7 (BioStatus, Loughborough, UK, DR71000): 0.3 μM, TMRE: tetramethylrhodamine ethyl ester (Thermo Fisher Scientific, T669): 200 nM, FeRhoNox-1 (Goryo Chemical, Sapporo, Hokkaido, Japan, GC901): 10 μM, MitoSOX Red (Thermo Fisher Scientific, M36008): 5 μM, erastin (Selleckchem, Houston, TX, USA, S7242): 10 and 20 μM, CCCP: carbonyl cyanide 3-chlorophenylhydrazone (Sigma, St. Louis, MO, USA, Cat#C2759), Nec-1s (Calbiochem, San Diego, CA, USA, 480065): 10 μM, Fer1 (Xcess Biosciences, Chicago, IL, USA, 053224): 1 μM, DFO (Sigma-Aldrich, St. Louis, MI, USA, D-9533): 50 μM, and CPX (Sigma-Aldrich, C0415): 5 μM. Z-VAD-FMK (Bachem, Bubendorf, Switzerland, N-1510), a caspase peptide inhibitor, was used at a concentration of 10 μM, while DEVD-AMC (Pepta Nova, Sandhausen, Germany, 3171-V), a fluorogenic substrate for caspase-3, was used at 20 μM. The mitochondrial complexes inhibitors rotenone (Sigma, Cat#R8875), antimycin A (Sigma, Cat#A8674), and oligomycin (Sigma, Cat#75351) were used at 0.5 and 10 μM, 0.5 and 50 μM, and 1.5 μM, respectively. Hemin (Sigma-Aldrich, H9039) was used at 5 and 10 μM, zinc protoporphyrin: ZnPP (Enzo Life Sciences, ALX-430-049-M025) was used at 1 μM, and ferrous ammonium sulfate: Fe(NH_4_)_2_(SO_4_)_2_·6H_2_O (Sigma-Aldrich, FX0245) was used at 1 mM. Trimethylsilyldiazomethane (Sigma-Aldrich, 362832): a 2.0 M solution in hexanes was used. Toluene, methanol, *n*-hexane, and diethyl ether were purchased from Chem-Lab NV (Zedelgem, Belgium, HPLC grade).

### 2.2. Nemorosone Isolation and Characterization. Synthesis and Characterization of Methylated Derivative

TLC was performed using precoated silica gel plates (Macherey-Nagel SIL G-25 UV_254_). Chemical shifts for the ^1^H NMR and ^13^C NMR spectra, recorded on a Bruker Avance 400 spectrometer, were reported in parts per million with reference to the residual solvent signal (CDCl_3_: 7.26 ppm; CD_3_OD: 3.30 ppm and 49.00 ppm). Coupling constants (J) are expressed in hertz. Electrospray mass spectra were recorded by means of an Agilent 1100 series single quadrupole MS detector type VL, with APCI and API-ES sources, and provided with a Phenomenex Luna C18 (2) column (5 µm 250 mm × 4.60 mm). An Agilent 1100 series connected to a 6220A TOF-MS detector, equipped with an APCI-ESI multi-mode source, was used to conduct high resolution mass spectrometry (HRMS). A Perkin-Elmer 1000 FT-IR infrared spectrometer (HATR) was utilized to record the infrared spectra. A Perkin Elmer 241 polarimeter was used to measure optical rotation.

Nemorosone was isolated, as previously described [2], from the floral resin of *Clusia rosea*. Concisely, an EtOH-H_2_O solution was used to crystallize nemorosone from the resin of the flowers of this plant species. Figure S1A shows the structure of nemorosone: C_33_H_42_O_4_, a mixture of tautomers (1*S*,5*R*,7*R*)-5-benzoyl-4-hydroxy-6,6-dimethyl-1,3,7-tris(3-methylbut-2-en-1-yl)bicyclo [3.3.1]non-3-ene-2,9-dione and (1*S*,5*S*,7*R*)-1-benzoyl-4-hydroxy-8,8-dimethyl-3,5,7-tris(3-methylbut-2-en-1-yl)bicyclo[3.3.1]non-3-ene-2,9-dione.

Purity of isolated nemorosone was >99%, as determined via reversed-phase HPLC, with detection at 214/254 nm (retention time: 6.9 min; see Figure S4). Eluting conditions: eluent A/eluent B (50/50) for 30 s, followed by gradient elution (A/B from 50/50 to 0/100) over 6 min (eluent A: 0.1% HCOOH in water; eluent B: acetonitrile) on a Phenomenex Luna C18 (2) column (5 µm 250 mm × 4.60 mm). Analytical data are in agreement with the literature and identical to recorded data of commercially available nemorosone (purchased from Cayman Chemical, Ann Arbor, MI, USA, 24256). Rf 0.22 in hexane/EtOAc 8/2 [Lit: ≈0.21 [25]]. ^1^H NMR (CD_3_OD, 400 MHz): 7.53 (br d, *J* = 7.7 Hz, 2H), 7.43 (app tt, *J* = 7.4 Hz/1.2 Hz, 1H), 7.22–7.28 (m, 2H), 4.96–5.11 (m, 3H), 3.13 (dd, *J* = 15.0 Hz/7.8 Hz, 1H, A-part of ABX-system), 3.07 (dd, *J* = 14.8 Hz/7.4 Hz, 1H, B-part of ABX-system), 2.54 (dd, *J* = 14.7 Hz/7.8 Hz, 1H, A-part of ABX-system), 2.47 (dd, *J* = 14.5 Hz/7.3 Hz, 1H, B-part of ABX-system), 2.09–2.19 (m, 1H), 2.01 (br dd, *J* = 13.1 Hz/2.7 Hz, 1H), 1.65–1.85 (m, 2H), 1.68 (s, 3H), 1.64 (app s, 12H), 1.58 (s, 3H), 1.39–1.50 (m, 1H), 1.33 (s, 3H), 1.10 (s, 3H) ppm. ^13^C NMR (CD_3_OD, 100 MHz): 209.26 (C), 194.88 (C), 138.23 (C), 135.25 (C), 134.24 (C), 133.66 (C), 133.12 (CH), 129.61 (CH), 128.78 (CH), 123.99 (CH), 122.12 (CH), 121.18 (C), 120.79 (CH), 44.64 (CH), 30.40 (CH_2_), 28.19 (CH_2_), 26.30 (CH_3_), 26.02 (CH_3_), 24.35 (CH_3_), 22.32 (CH_2_), 18.30 (CH_3_), 18.11 (CH_3_), 17.97 (CH_3_), 16.20 (CH_3_) ppm. [a]_D_^20^ +99° (c 0.095, MeOH) [Lit: −98.3° (ent-nemorosone, c 1.19, MeOH) [26]]. IR (HATR): 3534 (m), 3418 (m), 2966 (m), 2916 (m), 2882 (m), 1711 (s), 1699 (s), 1582 (vs), 1446 (m), 1391 (m), 1368 (vs), 1317 (m), 1262 (m), 1242 (m), 1215 (s), 1197 (m), 1185 (m), 1172 (m), 1157 (m), 1128 (m), 1101 (m), 1063 (m), 1032 (w), 1019 (m), 1003 (w), 959 (w), 936 (w), 922 (w), 897 (w), 839 (s), 799 (m), 772 (w), 751 (m), 730 (w), 690 (m), 666 (m) cm^−1^. HRMS (ESI, positive mode): calculated for C_33_H_43_O_4_^+^ [M+H^+^]: 503.3156, found: 503.3149. Data on isolated natural nemorosone: [2]; data on synthetic ent-nemorosone: [26]; data on synthetic racemic nemorosone: [25]. Copies of ^1^H and ^13^C (APT) spectra can be found in Supplementary Materials (Figures S5 and S6, respectively).

For the synthesis of *O*-methylated nemorosone, to a solution of nemorosone (200 mg, 0.398 mmol, 1 eq) in toluene/methanol (10 mL, 4/1), trimethylsilyldiazomethane (1.2 mL, 2.0 M in hexanes, 2.4 mmol, 6 eq) was added dropwise. After stirring the reaction mixture (a pale yellow solution) at room temperature for 30 min, silica was added to quench the excess of reagent. The resulting suspension was filtered and, under reduced pressure, the filtrate was concentrated. The ^1^H NMR spectrum of the crude mixture of both formed isomers was consistent with findings in the literature data on pure individual compounds [25]; integration of diagnostic methyl ester signals showed an isomer ratio of 78/22 (see Figure S7). The residue was partially purified using flash chromatography (gradient elution: hexane/ether 99/1–93/7), affording pure major isomer (48 mg, 0.093 mmol, 23% yield) and a mixture of major and minor isomers (153 mg, 0.296 mmol, 74% yield, ratio major/minor: 72.4/27.6, as determined via RP-HPLC/MS, integration of peaks at 214 nm, retention times 6.3 min and 6.5 min; see Figure S8). Eluting conditions: eluent A/eluent B (100/0) during 30 s, followed by gradient elution (A/B from 100/0 to 0/100) over 6 min (eluent A: 5 mM NH_4_OAc in water; eluent B: acetonitrile) on a Phenomenex Luna C18 (2) column (5 µm 250 mm × 4.60 mm). Major isomer: Rf 0.25 in hexane/ether 9/1. HRMS (ESI, positive mode): calculated for C_34_H_45_O_4_^+^ [M + H^+^]: 517.3312, found: 517.3335. Minor isomer: Rf 0.17 in hexane/ether 9/1. HRMS (ESI, positive mode): calculated for C_34_H_45_O_4_^+^ [M + H^+^]: 517.3312, found: 517.3330.

### 2.3. Conditions for Cell Culture

DMEM medium supplemented with 10% (*v*/*v*) fetal calf serum (FCS), sodium pyruvate (1 mM), l-glutamine (1 mM), and non-essential amino acids (1 mM) was used to cultivate U87MG and U373MG human glioblastoma cells and HT22 cells (non-tumorigenic mouse hippocampal neuronal cell line); while IMR-32 (human neuroblastoma cell line) and HT1080 human fibrosarcoma cells were cultured in RPMI 1640 and EMEM medium, respectively, both supplemented in the same way as DMEM medium. Each cell line was obtained from ATCC. Every 3–4 days, cells cultures were split using a trypsin/EDTA solution and maintained at 37 °C in a humid 5% CO_2_ environment. It is important to highlight that these cancer cell lines were chosen both for their clinical relevance (they are representative cell lines of tumors refractory to conventional therapy) and for their reported sensitivity to the induction of non-apoptotic regulated cell death [17,19,27,28]. In addition, nemorosone had not been tested in any of these cell lines.

### 2.4. Analysis of Cell Death and Caspase-3 Activity

Using the FLUOstar Omega fluorescence plate reader (BMG Labtech GmbH), cell death and caspase-3 activity were measured as previously reported [29,30]. Briefly, cells were seeded in a 96-well plate, and all experiments were carried out in triplicate. The following day, after being preincubated with the selected inhibitors, cells were treated with stimuli at desired concentrations in the presence of SytoxGreen and DEVD-AMC. At 1 h intervals, the fluorescence intensity of both fluorescent probes was measured. Percent cell death was calculated using Triton X-100 (0.05%) as a reference for 100% cell death. Following this same procedure, live cell images of seeded cells were obtained using a Zeiss LSM780 confocal microscope. The ImageJ program was used to merge the images. To analyze the induction of cell death, SytoxBlue staining was also used in conjunction with flow cytometry (BD LSR-Fortessa, BD Biosciences, Franklin Lakes, NJ, USA).

### 2.5. Lipid ROS Analysis

Lipid ROS generation was determined by a previously described methodology [30]. In short, in a 6-well plate, HT1080 (300,000 cells/well) and IMR-32 (500,000 cells/well) cells were seeded. Cells were stimulated the following day and harvested. Fluorescent probes, C11-BODIPY and DRAQ7, were added to the wells 10 min prior to each time point, and lipid ROS accumulation was measured by flow cytometry (BD LSRFortessa, BD Biosciences). B530 (C11-BODIPY) and R780 (DRAQ7) channels were used to measure fluorescence. Only non-permeable live cell fluorescence was evaluated. Per condition, a minimum of 10,000 cells were examined.

### 2.6. Mitochondrial ROS Analysis

Mitochondrial ROS generation was determined using MitoSOX Red. In brief, HT1080 cells (300,000 cells/well) were seeded in a 6-well plate and incubated overnight. Afterward, cells were exposed to the test compounds according to the instructions of the experiment. After being washed with pre-warmed HBSS (Thermo Fisher Scientific, 14025076), cells were then incubated with fresh medium containing MitoSox Red for 15 min at 37 °C. Subsequently, cells were washed with HBSS and collected in PBS (Thermo Fisher Scientific, 10010023) containing SytoxBlue for measurement using BD FACSVerse (BD Biosciences). B586 (MitoSOX Red) and V448 (SytoxBlue) channels were used to measure fluorescence. Only non-permeable live cell fluorescence was evaluated. Per condition, a minimum of 10,000 cells were examined.

### 2.7. Determination of Cellular Labile Fe^2+^ Pool

FeRhoNox-1 dye was used to measure iron levels as previously described [17]. HT1080 cells (300,000 cells/well) were seeded in a 6-well plate and incubated overnight. Cells were harvested the next day and centrifuged at 300× *g* for 5 min. Then, cells were centrifuged at 300× *g* for 5 min after being washed with PBS buffer. The collected cells were stained with FeRhoNox-1 in PBS and kept in a CO_2_ incubator for 30 min. Following HBSS washing, cell culture was dissolved in 300 μL of HBSS containing SytoxBlue, and examined using BD LSRFortessa (BD Biosciences). Y585 (FeRhoNox-1) and V450 (SytoxBlue) channels were used to measure fluorescence. Only non-permeable live cell fluorescence was evaluated. Per condition, a minimum of 10,000 cells were examined.

### 2.8. Measurement of Mitochondrial Membrane Potential

In a 6-well plate, HT1080 (300,000 cells/well) and IMR-32 (500,000 cells/well) cells were seeded. The following day, after cells had received the indicated treatment, TMRE (200 nM) was added, and the mixture was incubated for 30 min. The cells were washed with PBS to remove extra TMRE before being collected for analysis with the BD LSRFortessa (BD Biosciences). B575 (TMRE) and V450 (SytoxBlue) channels were used to measure fluorescence. Only non-permeable live cell fluorescence was evaluated. Per condition, a minimum of 10,000 cells were examined.

### 2.9. Measurement of GSH Levels

As previously reported [17], glutathione levels were determined using QuantiChrom Glutathione Assay Kit (BioAssay Systems, Hayward, CA, USA, DIGT-250). Concisely, 1,000,000 cells per condition (HT1080 or IMR-32 cells) were seeded in a 6-well plate. The next day, cells were treated as indicated in each experiment. After that, cells were gathered, transferred to a new tube, and centrifuged at 425× *g* for 5 min at 4 °C. After being resuspended in 300 μL of PBS, each cell pellet was lysed using ultrasound. Each lysate was centrifuged at 14,000 rpm for 10 min at 4 °C. The cleared lysate was then used to calculate the amount of GSH present in each sample following the kit descriptions.

### 2.10. Measurement of Intracellular Glutamate Levels

Intracellular glutamate levels were measured using Amplex^®^ Red Glutamic Acid/Glutamate Oxidase Assay Kit (Thermo Fisher Scientific, A12221). HT1080 cells (400,000 cells/well) were seeded in a 6-well plate and incubated overnight. The following day, cells were treated according to the conditions described in each experiment and collected by centrifugation at 300× *g* for 5 min. After removing the supernatant, the pellet was resuspended in PBS buffer. Afterward, each sample was centrifuged again at 300× *g* for 5 min, and the pellet was resuspended in 100 μL of Tris HCl buffer (0.1 M, pH = 7.5). Then, cells were lysed by sonication, and each sample was diluted (2×) with Tris HCl buffer. Next, 50 μL of the diluted samples were transferred into separate wells of a microplate (OptiPlate 96-well plate), and the amount of intracellular glutamate was calculated following the kit descriptions.

### 2.11. Measurement of ATP Levels

ATP levels were determined using CellTiter-Glo 2.0 Assay Kit (Promega, Madison, WI, USA, Cat# G9242/3) based on the firefly luciferin–luciferase assay system. Briefly, HT1080 cells (400,000 cells/well) were seeded in a 96-well plate in the absence (control) or presence of nemorosone, CCCP, or oligomycin, in line with the conditions described in the experiment legend. The measurement was performed in accordance with the instructions of the kit.

### 2.12. Measurement of Mitochondrial Respiration in Intact Cells

Intact HT1080 or IMR-32 cells were added to a 2 mL chamber at a concentration of 1,000,000 cells/mL. Oxygen consumption was measured at 37 °C using a high-resolution respirometer (Oxygraph-2k Oroboros Instruments, Innsbruck, Austria). Oxygen flow per cell (pmol·s^−1^·mL^−1^) was recorded continuously using DatLab software 6 (Oroboros Instruments). After approximately 10 min of monitoring oxygen consumption, corresponding sequential injections of selected compounds and inhibitors were performed as indicated by the phosphorylation control protocol [31].

### 2.13. OCR and ECAR Measurement

The Seahorse XFe96 Analyzer (Agilent) was used to measure the oxygen consumption rate (OCR) and extracellular acidification rate (ECAR). HT1080 cells (200,000 cells/well) were seeded into 96-well plates and incubated for 24 h. Before the assay, the culture medium was changed to a similar medium without phenol red and with 25 mM glucose, 1 mM sodium pyruvate, and 1 mM glutamine, and the cells were equilibrated for 30 min at 37 °C. During the assay, the compounds of interest were added, and the OCR and ECAR values were measured at intervals of approximately 6 min.

### 2.14. RNA Sequencing and Data Analysis

An RNA 6000 nano chip (Agilent Technologies, Santa Clara, CA, USA) as well as an RNA labchip (Caliper GX-Perkin Elmer) were used to assess the total RNA quality of gall and control samples. Concentrations were determined using a Quant-it Ribogreen RNA assay (Life Technologies). Then, 265 ng of RNA were employed for the library prep through the QuantSeq 3′ mRNA libr prep FWD kit (Lexogen). Library prep was carried out in accordance with the recommendations of the manufacturer. In brief, first-strand cDNA synthesis was performed, followed by an RNA removal step. Then, second-strand synthesis was performed with the use of UMIs, after which the cDNA was purified using beads (Lexogen). In addition to being purified with beads, the cDNA was used for 13 cycles of enrichment PCR. Using a high sensitivity DNA chip from Agilent Technologies, the quality was examined. To enable equimolar library pooling, a qPCR assay was used to quantify the libraries in accordance with the Illumina protocol. Finally, sequencing was carried out on a Nextseq500 using 20% Phix spike-in (single-end reads, 76 cycles).

Through the use of FastQC (version 0.11.9), the quality of the reads was confirmed [32]. The following parameters were used to trim reads with Trimmomatic (version 0.39): ILLUMINACLIP:<TruSeq3-SE adapter file>:3:30:10, SLIDINGWINDOW:5:20, MINLEN:20 [33]. STAR (version 2.7.8a) was used for mapping with the subsequent parameters: readFilesCommand zcat, outFilterMultimapNmax 1 and outSAMtype BAM SortedByCoordinate using the GRCh38.106 genome build [34].

These R packages were utilized to create a count table: GenomicFeatures (version 1.44.2), to convert the GRCh38.106 GTF file into a Granges object, and GenomicAlignments (version 1.28.0), for the summarizeOverlaps function to create the count table [35]. The counting options were as follows: mode = ‘Union’, singleEnd = TRUE, and ignore.strand = FALSE. To find differentially expressed genes, DESeq2 (version 1.32.0) was used with a Benjamini–Hochberg FDR cutoff of 0.05 [36]. Lists of differentially expressed genes were used for downstream analysis using Ingenuity pathway analysis (Qiagen). An R (v 4.1.3) environment was used for the analysis.

### 2.15. RNA Isolation and Analysis by RT-qPCR

Total RNA from treated HT1080 cells was extracted according to the NucleoSpin^®^ RNA Plus protocol (fifth revision, corresponding to January 2021) prepared by MACHEREY-NAGEL GmbH & Co. KG (Düren, Germany). For DNA synthesis, a C1000 Touch^®^ thermocycler (Bio-Rad) was used. The qPCR analysis was performed under the following conditions: 95 °C for denaturation, 60 °C for hybridization, and 70 °C for elongation. qbase+ software (Biogazelle) was used to calculate the expression levels of mRNA (HMOX1: Bio-Rad, Hercules, CA, USA, qHsaCIP0033307) from the structural genes (housekeeping genes), HMBS (Bio-Rad, qHsaCID0038839) and RPL3 (Bio-Rad, qHsaCED0038656), which were used as internal references.

### 2.16. Protein Extraction and Western Blot Analysis

At designated times, test compound-treated HT1080 cells were harvested and subjected to two washes with cold PBS solution. A cell lysis buffer (Cell Signaling Technology, Danvers, MA, USA) was used to extract the total cytosolic proteins, and their concentrations were determined by the Bradford method. In the wells of the 10% SDS-PAGE gel, 25 µg of protein were loaded along with the molecular weight marker. After performing the run (1 h, 100 V), the transfer of the proteins from the gel to nitrocellulose membranes was carried out. Subsequently, the membranes were blocked with 5% skim milk powder prepared in TBST saline (0.05% Tween 20). The membranes were incubated for 24 h at 4 °C with each primary antibody of interest (except in the case of β-tubulin, with which they were incubated for 1 h). Peroxidase-labeled secondary antibodies (PerkinElmer Life Sciences) were used to detect immunoreactive proteins.

### 2.17. Statistical Analysis

Unpaired Student’s *t*-test was carried out, using GraphPad Prism version 9.2.0 (GraphPad Software, San Diego, CA, USA), to calculate *p* values (* *p* < 0.05, ** *p* < 0.01, *** *p* < 0.001, **** *p* < 0.0001; see figure legends for more information), with the exception of Figure 3C, where a two-way ANOVA test was employed. Unless otherwise stated, data are displayed as the mean ± SD of three separate experiments.

## 3. Results

### 3.1. Nemorosone Is Highly Cytotoxic in Fibrosarcoma HT1080 Cells through Induction of Ferroptosis

**Figure 1:**
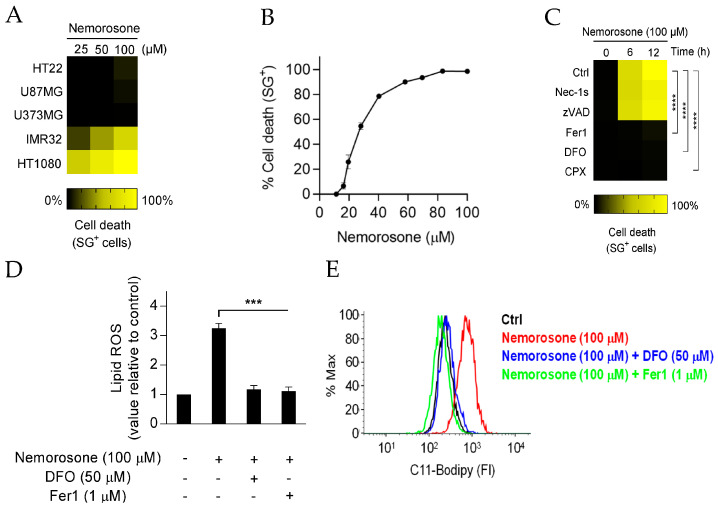
High cytotoxicity of nemorosone in human fibrosarcoma cell line HT1080 is due to ferroptosis induction. (**A**) Heatmap showing the sensitivity to cell death of different cancer and non-tumorigenic cell lines after exposure to various concentrations of nemorosone: HT22 (mouse hippocampal neuronal cell line), U87MG and U373MG (human glioblastoma cell lines), IMR-32 (human neuroblastoma cell line), and HT1080 (human fibrosarcoma cell line). Cell lines were incubated with nemorosone for 24 h. (**B**) Cytotoxic dose-response curve in HT1080 cells 12 h after nemorosone treatment. (**C**) Heatmap showing the sensitivity to cell death of HT1080 cells after being exposed to 100 μM of nemorosone, with or without the following inhibitors: the RIPK1-kinase inhibitor necrostatin-1 (Nec-1s, 10 μM), the pan-caspase inhibitor Z-VAD-FMK (10 μM), and the ferroptosis inhibitors ferrostatin-1 (Fer1, 1 μM), deferoxamine (DFO, 50 μM), and ciclopirox olamine (CPX, 5 μM). (**D**) Analysis using flow cytometry of the C11-BODIPY lipid peroxidation sensor in live HT1080 cells (DRAQ7-negative cells) following nemorosone treatment (100 μM, 4 h). Ferroptosis inhibitors: DFO (50 μM) and Fer1 (1 μM). (**E**) Flow cytometry analysis (histogram) of the lipid peroxidation sensor (C11-BODIPY-581/591 dye) on live-gated HT1080 cells (DRAQ7-negative cells) after treatment with nemorosone (100 μM, 4 h) and its combination with the ferroptosis inhibitors DFO (50 μM) and Fer1 (1 μM). FI is the fluorescent intensity. Traces are representative of three independent experiments. All quantitative data are shown as mean ± SD from three separate experiments. *** *p* < 0.001 and **** *p* < 0.0001 determined by unpaired Student’s *t*-test.

First, we analyzed the cytotoxic effect of nemorosone (Supplemental Figure S1A) in a panel of cancer cell lines. Nemorosone was highly potent in killing HT1080 fibrosarcoma cell lines and high-risk MYCN-amplified IMR-32 neuroblastoma cells (Figure 1A). The most potent effect was exerted on HT1080 cells, reaching 100% of cell death in 12 h (Figure 1A,B and Figure S1B), while in IMR-32 cells around 70% of cell death was reached in 24 h (Figure 1A). However, nemorosone did not show any cytotoxic effect in glioblastoma (U87MG and U373MG) and the non-tumorigenic mouse neuronal cell lines (HT22) after 24h (Figure 1A). The EC_50_ (half-maximal effective concentration) of nemorosone on HT1080 cells was determined to be 26.9 μM at 12 h (Figure 1B) and 16.7 μM at 24 h.

To determine the type of cell death induced by nemorosone and prior to nemorosone exposure, HT1080 and IMR-32 cells were treated with a variety of apoptotic and non-apoptotic cell death inhibitors. The cell death induced by nemorosone was prevented by the tested ferroptosis inhibitors, the iron chelators deferoxamine (DFO) and ciclopirox olamine (CPX) and the lipophilic radical trap ferrostatin-1 (Fer1) (Figure 1C and Figure S1C), but it was not affected by the pan-caspase inhibitor Z-VAD-FMK and the RIPK1-kinase inhibitor necrostatin-1 (Nec-1s) (Figure 1C and Figure S1C). Moreover, analysis of caspase-3 activity with fluorescent caspase-activity probe (DEVD-AMC) did not show any caspase activity in HT1080 or IMR-32 cells.

Considering that ferroptosis is characterized by high levels of lipid peroxidation compared to other cell death modalities [20], we examined this parameter after challenging HT1080 and IMR-32 cells with nemorosone using the fluorochrome C11-BODIPY. We observed that nemorosone triggers a time-dependent increase in lipid peroxidation in both fibrosarcoma and neuroblastoma cells (Figure 1D,E, Figure 3D and Figure S1D). Moreover, we noticed that DFO and Fer1 completely prevented the production of lipid hydroperoxide (Figure 1D,E and Figure S1D).

### 3.2. Nemorosone Acts as a Natural Class I Ferroptosis-Inducing Compound

Inactivation of GPX4 is one of the canonical ways of induction of ferroptosis and is exerted by class II ferroptosis inducers. Depletion of intracellular glutathione, which acts as a cofactor for GPX4 to reduce lipid hydroperoxides, is a second canonical way of ferroptosis induction, for example, by blocking the System xc cystine/glutamate antiporter by class I ferroptosis inducers [19,22,37,38,39,40].

**Figure 2:**
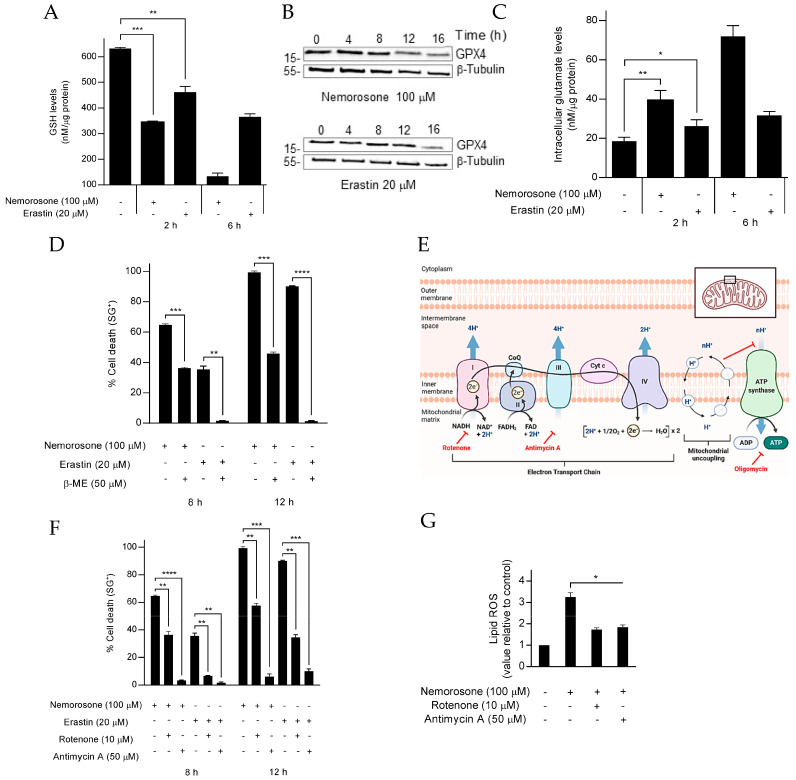
Nemorosone acts as a class I ferroptosis-inducing compound. (**A**) GSH levels in HT1080 cells treated with nemorosone (100 μM) or erastin (20 μM). (**B**) Western blot analysis of the expression of GPX4 and β-tubulin in HT1080 cells after treatment with nemorosone (100 μM) or erastin (20 μM). (**C**) Intracellular glutamate levels in HT1080 cells treated with nemorosone (100 μM) or erastin (20 μM). (**D**) Percentage of cell death at different time points induced by nemorosone (100 μM) or erastin (20 μM) in HT1080 cells, assessed using SytoxGreen dye, in the absence or presence of β-mercaptoethanol (β-ME, 50 μM). (**E**) Schematic representation of some inhibitors of the mitochondrial oxidative phosphorylation. Electrons from substrates pass through complexes I to IV of the electron transport chain. Protons (H^+^) are pumped into the intermembrane space using the energy generated by this process. The resulting proton gradient is used to drive ATP synthesis. This figure was created with BioRender.com (accessed on 17 July 2022). (**F**) Percentage of cell death induced by nemorosone (100 μM) and erastin (20 μM) in HT1080 cells, assessed using SytoxGreen dye, in absence or presence of the subsequent electron transport chain inhibitors: rotenone (10 μM) and antimycin A (50 μM). (**G**) Analysis using flow cytometry of the C11-BODIPY lipid peroxidation sensor in live HT1080 cells (DRAQ7-negative cells) following nemorosone treatment (100 μM, 4 h). Electron transport chain inhibitors: rotenone (10 μM) and antimycin A (50 μM). All quantitative data are shown as mean ± SD from three separate experiments. * *p* < 0.05, ** *p* < 0.01, *** *p* < 0.001, **** *p* < 0.0001 determined by unpaired Student’s *t*-test.

Similar to erastin, a class I FIN, we observed a substantial decrease in GSH levels in the fibrosarcoma and neuroblastoma cells after nemorosone treatment (Figure 2A and Figure S1E). We did not detect any GPX4 depletion at protein level after incubation with nemorosone or erastin, apart from the expected reduction in the signal due to the protein degradation exerted by the occurrence of cell death at the highest time points (Figure 2B). Consistent with the GSH levels’ decrease, we found that nemorosone, like erastin, increases the intracellular glutamate levels, suggesting the inhibition on the cystine/glutamate exchange mediated by the System xc cystine/glutamate antiporter (Figure 2C). If the blockade of cystine import through the System xc antiporter can trigger ferroptosis, then providing this metabolite to cells through an alternative means should rescue the cells from death [19]. Therefore, we pretreated cells with β-mercaptoethanol (β-ME), which reduces extracellular cystine to cysteine and bypasses the inhibition of the System xc antiporter, since cysteine can be imported via other pathways [41]. As shown in Figure 2D, β-ME inhibited nemorosone- and erastin-induced cell death in HT1080 cells, as previously reported [42]. However, β-ME inhibited 100% of the cell death induced by erastin, whereas it inhibited the cell death induced by nemorosone by approximately 60% (Figure 2D). The above results indicate not only that nemorosone partially acts as an erastin-like class I ferroptosis inducer but also that nemorosone additionally induces ferroptosis by another mechanism that circumvents the protection exerted by β-ME.

Considering the central role of mitochondria in erastin-induced ferroptosis [23], we evaluated the involvement of the Electron Transport Chain (ETC) in nemorosone-induced ferroptosis using the mitochondrial complex I inhibitor (rotenone) and the mitochondrial complex III inhibitor (antimycin A) (Figure 2E). We found that both inhibitors suppressed cell death and the lipid ROS accumulation triggered by nemorosone and erastin (Figure 2F,G). These results show that functional ETC are required for nemorosone to induce ferroptosis. Altogether, these results suggest that nemorosone acts as a class I FIN and as an ETC-dependent ferroptosis inducer.

### 3.3. Mitochondrial Uncoupling of Nemorosone Is Indispensable for Ferroptosis Induction

To examine whether the uncoupling effect of nemorosone is required to induce ferroptosis, we first verified its uncoupling potency compared to the classical protonophoric mitochondrial uncoupler CCCP by measuring the oxygen consumption rate (OCR) increase, the drop of mitochondrial membrane potential (MMP), ATP levels reduction, extracellular acidification rate (ECAR) increase (which can indicate higher rates of glycolysis), and mitochondrial ROS production (mitoROS). We found that nemorosone is at least an equally potent uncoupler compared to CCCP in both the neuroblastoma (Figure S1F–H) and fibrosarcoma contexts (Figure S2A–G).

**Figure 3:**
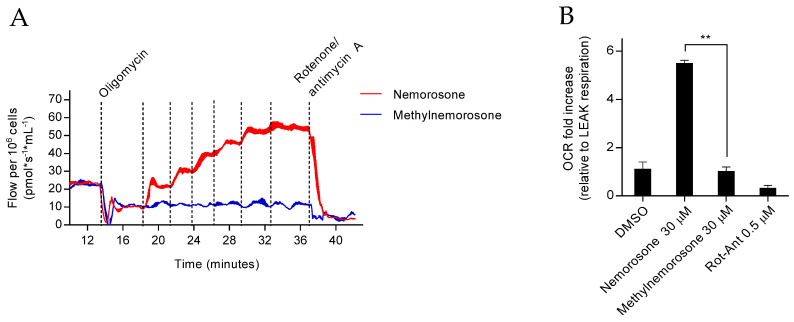

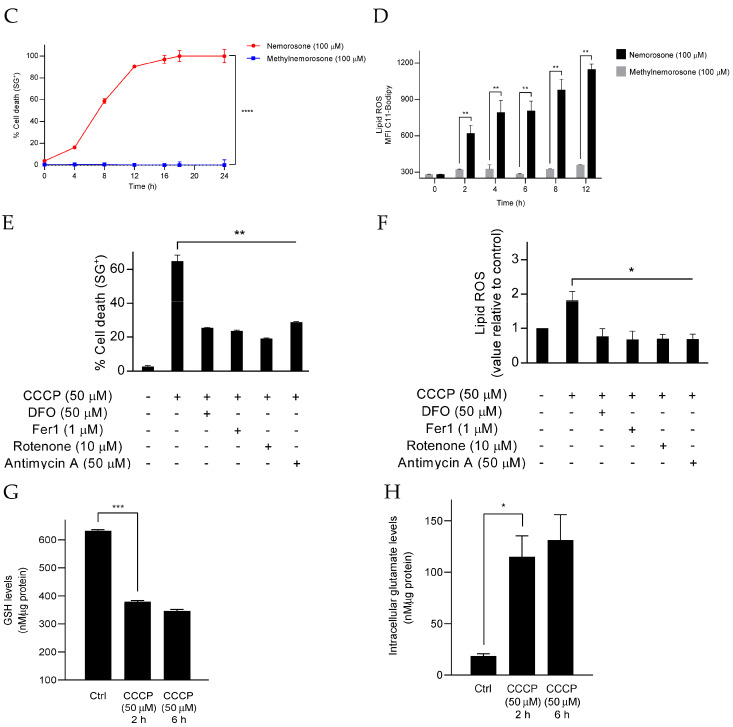
Nemorosone-induced ferroptosis may be associated with mitochondrial uncoupling. (**A**) Phosphorylation control protocol performed by high-resolution respirometry in intact HT1080 cells (1,000,000 cells/mL). After the addition of oligomycin (1.5 μM), cells were titrated with nemorosone and methylnemorosone in steps of 5 μM additions, totalizing 30 μM of both compounds in the Oxygraph-2k chambers (2 mL). DMSO was added, similar to nemorosone. The O_2_ flow (pmol·s^−1^·mL^−1^) was inhibited to a constant level after the addition of rotenone–antimycin A mix (0.5 μM). Every addition is represented by vertical dot lines. (**B**) Oxygen consumption rate (OCR) fold increase was relative to LEAK state (reached after the inhibition of F_1_F_O_ ATP synthase by oligomycin) in HT1080 cells exerted by the separated addition of DMSO (30 μM), nemorosone (30 μM), methylnemorosone (30 μM), and rotenone–antimycin A mix (Rot-Ant, 0.5 μM). (**C**) Percentage of cell death at different time points induced by nemorosone (100 μM) and methylnemorosone (100 μM) in HT1080 cells, assessed using SytoxGreen dye. (**D**) Analysis using flow cytometry of the C11-BODIPY lipid peroxidation sensor in live HT1080 cells (DRAQ7-negative cells) following nemorosone (100 μM) or methylnemorosone (100 μM) treatment at different time points. MFI is the median fluorescence intensity of the fluorophore. (**E**) Percentage of cell death induced by CCCP (12 h, 50 μM) in HT1080 cells, assessed using SytoxBlue dye. Ferroptosis inhibitors: DFO (50 µM) and Fer1 (1 µM). Electron transport chain inhibitors: rotenone (10 µM) and antimycin A (50 µM). (**F**) Analysis using flow cytometry of the C11-BODIPY lipid peroxidation sensor in live HT1080 cells (SytoxBlue-negative cells) following CCCP treatment (12 h, 50 µM). Ferroptosis inhibitors: DFO (50 µM) and Fer1 (1 µM). Electron transport chain inhibitors: rotenone (10 µM) and antimycin A (50 µM). (**G**) GSH levels in HT1080 cells after treatment with CCCP (50 μM) during 2 h and 6 h. (**H**) Intracellular glutamate levels in HT1080 cells after treatment with CCCP (50 μM) during 2 h and 6 h. All quantitative data are shown as mean ± SD from three separate experiments. * *p* < 0.05, ** *p* < 0.01, *** *p* < 0.001, and **** *p* < 0.0001 determined by unpaired Student’s *t*-test (**B**,**D**–**H**) and by two-way ANOVA test (**C**).

Next, we prepared an analogue of nemorosone by a reaction of the vinylogous carboxylic acid moiety with trimethylsilydiazomethane, giving *O*-methylated nemorosone (hereafter named “methylnemorosone”, isolated as a ~3/1 mixture of isomers resulting from both tautomeric forms of nemorosone), which lacks the uncoupling effect (Figure S2H and Figure 3A,B). We identified that, in the absence of its mitochondrial uncoupling effect, nemorosone shows no more cytotoxic effect nor lipid peroxidation increase in HT1080 cells (Figure 3C,D). These results indicate, for the first time, the reactive moiety that is crucial for nemorosone-induced protonophoric mitochondrial uncoupling and ferroptosis induction. Furthermore, this association between nemorosone-induced ferroptosis and mitochondrial uncoupling is consistent with the previously stated requirement of nemorosone dependency on a functional ETC to trigger ferroptosis.

Finally, we checked whether CCCP could also induce ferroptotic cell death. We found that both CCCP-induced cell death and lipid peroxidation were inhibited by canonical inhibitors of ferroptosis (Fer1 and DFO) and by ETC inhibitors (rotenone at complex I and antimycin A at complex III) (Figure 3E,F). Moreover, we observed that CCCP decreases GSH levels, which is associated, similar to nemorosone, with an increased level of intracellular glutamate (Figure 3G,H). These results show, for the first time, the capacity of not only nemorosone but also CCCP to induce ferroptosis, indicating that other protonophoric uncouplers could induce ferroptotic cell death.

### 3.4. Nemorosone-Induced Ferroptosis Involves Excessive Activation of Heme Oxygenase-1

**Figure 4:**
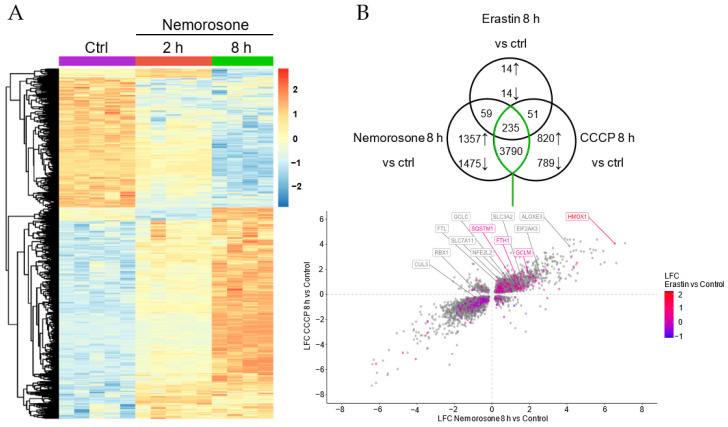

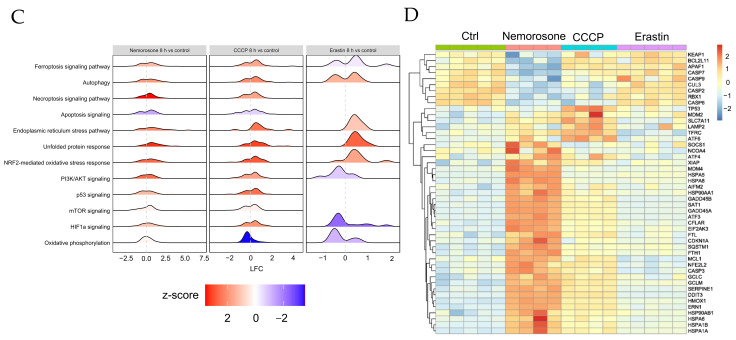
Genes and signaling pathways differentially expressed after treatment with nemorosone, CCCP, and erastin in HT1080 cells. (**A**) Heatmap of the expression of genes that are differentially expressed after both nemorosone (100 μM) 2 h and nemorosone (100 μM) 8 h treatments, compared to the control. Each column represents a sample; each row represents a gene. The genes have been clustered, and their expression has been centered and scaled. (**B**) Venn diagram showing differentially expressed (DE) genes, compared to the control, regulated by one, two, or all three treatments at the same time point (nemorosone 100 μM, CCCP 50 μM, erastin 20 μM; 8 h). The direction of the arrows indicates upregulation or downregulation in the case of individual treatments. Each intersection shows the set of genes modulated by two or three treatments. The scatterplot below was made by plotting the LFC values (log_2_fold change), metric indicating how much the expression of a gene changes compared to the control condition of the corresponding genes indicated by the green intersection: positive values indicate higher expression than the control, and negative values indicate lower expression than the control. DE genes by erastin are colored according to their LFC value. Known ferroptosis-related genes are labeled. (**C**) Ridge plots showing the IPA (Ingenuity Pathway Analysis) pathways that were found to be significantly enriched in DE gene sets. Each density plot shows the distribution of the LFC values of the DE genes involved in the enriched pathway. Density plots are scaled per panel. The color indicates the z-score, which is an IPA metric that predicts activation (positive values) or inhibition (negative values) of the pathway. (**D**) Heatmap of the expression of selected ferroptosis-related genes. Each column represents a sample; each row represents a gene. The genes have been clustered, and their expression has been centered and scaled.

To further characterize the mechanism of nemorosone-induced ferroptosis, we performed a genome-wide transcriptome analysis using RNA-Seq in HT1080 cells. We observed a significant transcriptional change after 2 and 8 h of nemorosone treatment (Figure 4A). Remarkably, we found that heme oxygenase-1 (HMOX1) was the most upregulated gene by nemorosone, and one of the most upregulated by CCCP and erastin, which is in line with both the NRF2 upregulation and downregulation of the components of the KEAP1–CUL3–RBX1 E3 ubiquitin ligase protein complex (Figure 4B,D, Table S1). HMOX1 expression is controlled by the transcription factor NRF2, which is kept in check by KEAP1-dependent degradative ubiquitination [43]. Correspondingly, one of the main signaling pathways induced by nemorosone, CCCP, and erastin, according to data analysis by Ingenuity Pathway Analysis (IPA), is the NRF2-mediated oxidative stress response (Figure 4C and Table S2).

**Figure 5:**
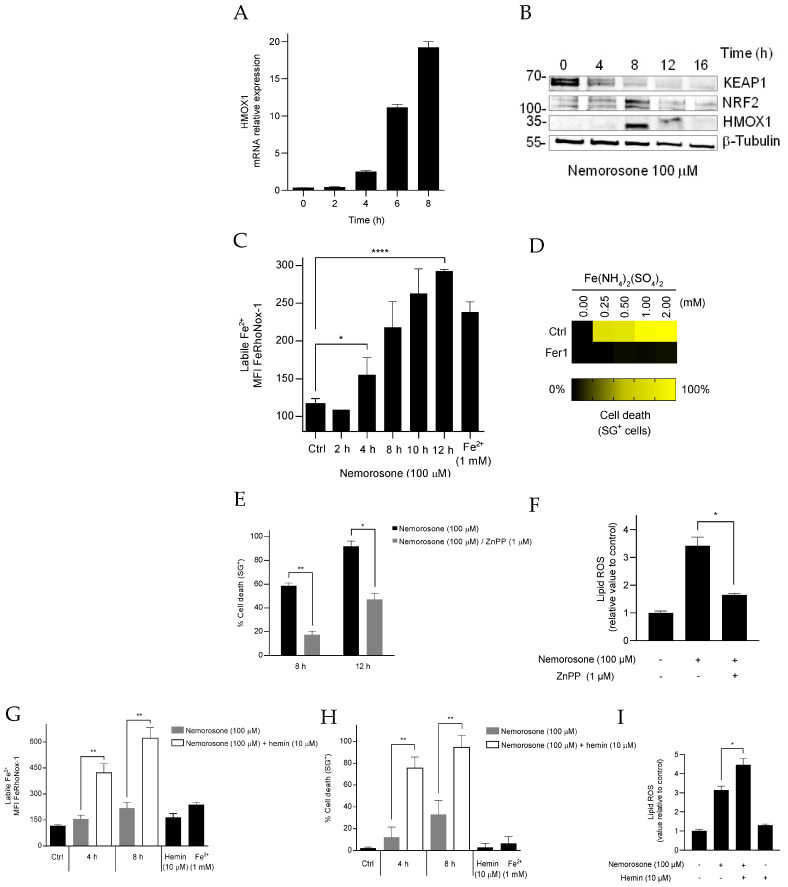

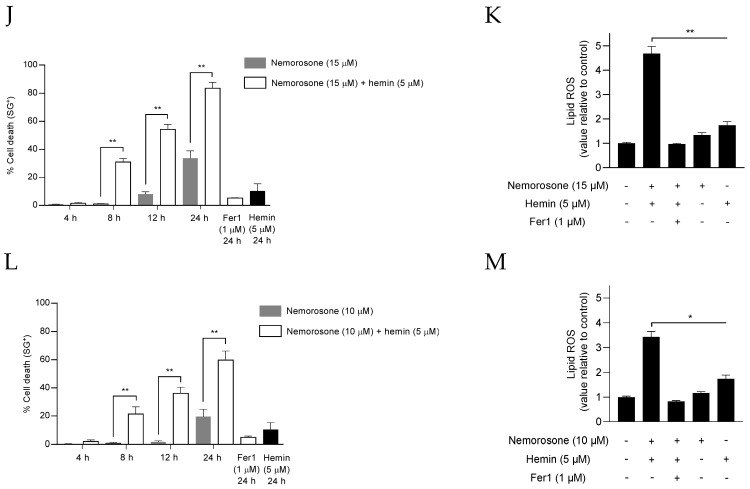
Nemorosone-induced ferroptosis in HT1080 cells involves excessive activation of HMOX1. (**A**) Relative *HMOX1* expression at mRNA level in HT1080 cells following nemorosone treatment (100 μM) at different time points. (**B**) Western blot showing KEAP1, NRF2, and HMOX1 modulation in HT1080 cells after treatment with nemorosone (100 μM). (**C**) Cellular levels of labile Fe^2+^, determined by flow cytometry using FeRhoNox-1 dye, at different time points, after nemorosone treatment (100 μM). Measurements were performed in live HT1080 cells (SytoxBlue-negative cells). Ferrous ammonium sulfate [Fe(NH_4_)_2_(SO_4_)_2_] was used as a control (2 h, 1 mM). MFI is the median fluorescence intensity of the fluorophore. (**D**) Heatmap showing the sensitivity to cell death of HT1080 cells treated with different concentrations of Fe(NH_4_)_2_(SO_4_)_2_ in the presence or absence of Fer1 (1 μM), assessed using SytoxGreen dye. (**E**) Percentage of cell death induced by nemorosone (100 μM) in the presence or absence of ZnPP (1 μM), assessed using SytoxGreen dye. (**F**) Analysis using flow cytometry of the C11-BODIPY lipid peroxidation sensor in live HT1080 cells (DRAQ7-negative cells) after 8 h of treatment with nemorosone (100 μM) in the presence or absence of ZnPP (1 μM). (**G**) Cellular levels of labile Fe^2+^, determined by flow cytometry using FeRhoNox-1 dye, in response to nemorosone (100 μM), in the presence or absence of hemin (10 μM). Measurements were performed in live HT1080 cells (SytoxBlue-negative cells). Ferrous ammonium sulfate was used as a control (2 h, 1 mM). MFI is the median fluorescence intensity of the fluorophore. (**H**) Percentage of cell death induced by nemorosone (100 μM) in the presence or absence of hemin (10 μM) in HT1080 cells, assessed using SytoxBlue dye. (**I**) Analysis using flow cytometry of the C11-BODIPY lipid peroxidation sensor in live HT1080 cells (DRAQ7-negative cells), following 8 h of treatment with nemorosone (100 μM), in the presence or absence of hemin (10 μM). (**J**,**L**) Percentage of cell death at different time points induced by nemorosone 15 and 10 μM, respectively, alone and in combination with hemin (5 μM). Cell death was assessed using SytoxGreen dye. (**K**,**M**) Analysis using flow cytometry of the C11-BODIPY lipid peroxidation sensor in live HT1080 cells (DRAQ7-negative cells) after treatment with nemorosone 15 and 10 μM, respectively, alone and in combination with hemin (5 μM) for 4 h. The combined results of 2 or 3 separate experiments are presented for (**C**–**M**). All quantitative data are shown as mean ± SD. * *p* < 0.05, ** *p* < 0.01, and **** *p* < 0.0001 determined by unpaired Student’s *t*-test.

Nemorosone-induced upregulation of HMOX1 was confirmed at both the mRNA (Figure 5A) and protein (Figure 5B) levels. Consistently, we found that when HMOX1 is upregulated, KEAP1 levels are reduced, while NRF2 levels are elevated (Figure 5B). The breakdown of heme molecules by HMOX1 is a major source of free Fe^2+^ [44]. In line with this, we observed a time-dependent increase in the intracellular levels of the labile iron pool (LIP) upon nemorosone treatment, measured using an Fe^2+^-selective probe (FeRhoNox-1) (Figure 5C). To check whether the increase in LIP is sufficient to induce ferroptosis in HT1080 cells, we treated cells with ferrous ammonium sulfate [Fe(NH_4_)_2_(SO_4_)_2_]. We revealed that the increase in LIP by the ferrous ammonium sulfate triggers ferroptotic cell death, which can be inhibited by Fer1 (Figure 5D). Of note, pharmacological inhibition of HMOX1 with zinc protoporphyrin (ZnPP), a metalloporphyrin that competitively inhibits the HMOX1 activity [45], prevents both lipid peroxidation and ferroptotic cell death after exposure to nemorosone (Figure 5E,F). Moreover, the combination of nemorosone with the HMOX1 substrate hemin increased labile Fe^2+^ levels, lipid peroxidation, and cell death (Figure 5G–I). These findings propose that nemorosone targets the KEAP1–NRF2–HMOX1 axis to promote ferroptosis by increasing the LIP through excessive activation of heme oxygenase-1. Therefore, it can also act as a class IV FIN [17].

To further confirm the role of HMOX1 upregulation in nemorosone-induced ferroptosis, we treated the cells with combinations of non-toxic concentrations of hemin and nemorosone. Interestingly, we observed that such a combination is sufficient to induce synergistically lipid peroxidation and cell death (Figure 5J–M).

## 4. Discussion

In the current work, nemorosone, a phytochemical isolated from the floral resin of the *C. rosea* plant, was identified to induce ferroptosis in fibrosarcoma and neuroblastoma cells. In 20 years of nemorosone anticancer-effect research, this is the first report that expands the potential of nemorosone by showing its capacity to induce another mechanism of cell death than apoptosis [4,11].

Notably, while nemorosone was cytotoxic against the neuroblastoma and fibrosarcoma cell lines, it did not show any effect on glioblastoma U87MG and U373MG cells. In general, the sensitivity to ferroptosis depends on the different endogenous mechanisms that protect cells against the lipid peroxidation that drives ferroptosis [46]. Recently, ferroptosis suppressor protein 1 (FSP1) was shown to play an essential role in the resistance of U373MG cells to erastin-induced System xc inhibition, while some unidentified mechanisms that support GPX4 function, independent of System xc activity, were also observed [47]. In line with this, a higher methionine uptake has been reported in gliomas than in normal astrocytes, which positively correlated with tumor viability and aggressiveness and indicated a greater reliance on transsulfuration, a metabolic pathway that connects methionine with glutathione biosynthesis independent of the System xc antiporter [48,49]. These reports allow us to explain a priori the resistance of glioblastoma cell lines to nemorosone-induced ferroptosis, although new experimental results are required to corroborate the aforementioned hypotheses. Conversely, based on the literature data, HT1080 and IMR-32 cells appear to be sensitive to the ferroptosis induced by decreased GSH levels through System xc inhibition and the increased labile iron pool (LIP) [17,19]. In this investigation, it was found that nemorosone induces ferroptosis by modulating these two parameters (Section 3.2 and Section 3.4).

On one hand, nemorosone-induced ferroptosis in HT1080 and IMR-32 cells involves the drop in the glutathione levels that can be associated with a blockade of the System xc cystine/glutamate antiporter or SLC7A11, which resembles the canonical ferroptosis-inducing pathway triggered by a class I FIN, such as erastin, also known as cysteine-deprivation-induced ferroptosis [19,23]. Inhibition of cystine import, which is required for GSH synthesis, results in depletion of intracellular GSH levels [16,33,37], an important cofactor for selenium-dependent GPX4. Therefore, GSH depletion by nemorosone could indirectly inactivate GPX4, leading to production of lipid ROS, which in turn results in lipid peroxidation and ferroptotic cell death [19,42]. It should be mentioned that more direct experimental approaches, such as the [^14^C]-cystine uptake assay [19], are required to confirm nemorosone-induced blockade of the System xc antiporter. Furthermore, inhibition of cystine entry is not necessarily the only pathway by which nemorosone could be lowering GSH levels. In fact, the decrease in the levels of NADPH, an electron-donor agent, relevant in the reduction of oxidized substrates, was reported as a common phenotypic effect for different structurally divergent uncoupling compounds [24]. It has been suggested that the dissipation of the mitochondrial membrane potential renders nicotinamide nucleotide transhydrogenase (NNT) incapable of maintaining the reduced NADPH state, which in turn can affect GSH regeneration via glutathione reductase (GR) [50,51]. In effect, the abundance of NADPH functions as a biomarker that is inversely correlated with the sensitivity of cells to the inducers of ferroptosis [52]. This important experimental issue should also be studied in future research.

On the other hand, nemorosone induces a non-canonical mechanism of ferroptosis by increasing the LIP, in response to the excessive activation of heme oxygenase-1 by targeting KEAP1 and NRF2, which is sufficient to trigger toxic lipid peroxidation. This result is comparable with the effect of withaferin A (WA), a natural FIN isolated from *Withania somnifera* roots, which at a medium dose induces ferroptosis through a massive upregulation of HMOX1 [17]. Likewise, Tagitinin C, another natural compound, induces ferroptosis in colorectal cancer cells through the PERK–NRF2–HMOX1 signaling pathway, and again the significant overexpression of HMOX1 led to the increase in the LIP, which promoted lipid peroxidation and ferroptosis [53]. As can be seen, these results with nemorosone add to a still small, but apparently growing, list of natural compounds that modulate the NRF2–HMOX1 axis to induce ferroptotic cell death, which could be related to some natural protection mechanism of plants (not yet reported) against pathogenic microorganisms. Similarly, HMOX1 was also shown as an essential enzyme that is involved in iron supplementation and lipid peroxidation in erastin-induced ferroptosis [54].

By giving cancerous cells antioxidant and cytoprotective effects and by removing toxic intracellular heme, the inducible intracellular enzyme HMOX1 was also shown to play a role in cancer progression [55]. This is in line with the fact that HMOX1 is elevated in various human malignancies such as, for example, fibrosarcoma tumors and HT1080 cells [56,57]. In consequence, HMOX1 inhibition was explored to reduce tumor growth [43,44,46]. Nevertheless, based on the current data, a massive activation of HMOX1 is important to kill HT1080 cells, strongly suggesting the efficacy of an opposite strategy: making tumor cells sensitive to the induction of ferroptosis via the therapeutic overactivation of HMOX1. At the same time, the active role of HMOX1 in tumor cells constitutes a significant difference compared to the healthy tissue and is, consequently, a way by which compounds such as nemorosone could induce a selective ferroptosis mechanism in cancer cells such as fibrosarcoma.

However, nemorosone-induced activation of the NRF2-mediated oxidative stress response pathway (Figure 4C), with the consequent modulation of NRF2 target genes (Table S1), shows that nemorosone activated the NRF2 pathway as an antioxidant and antiferroptotic response. It is the disproportionate upregulation of HMOX1 compared to other genes that results in a pro-ferroptotic effect. It is notable that *HMOX1* is the gene most upregulated by nemorosone: more than 90 times compared to the control, while *FTH1* is upregulated less than 3 times (Figure 4B and Table S1). That is, nemorosone generates an expression of *HMOX1* 30 times higher than the induced expression of *FTH1*, which, similar to what was reported for WA, suggests the induction of ferroptosis by raising the LIP in a context of insufficient ferritin buffering capacity [17]. In other words, the effect of nemorosone reveals a hormetic response associated with the NRF2–HMOX1 axis: a protective effect after moderate activation (classical and most common reports) vs. a cytotoxic effect after excessive activation (reported for some naturally occurring ferroptosis-inducing compounds).

It remains to be answered why nemorosone and other natural compounds generate such an overactivation of heme oxygenase-1. First of all, it must be taken into account that activation of HMOX1 by pathways other than NRF2 cannot be excluded. Several classes of stress-responsive transcription factors that activate *HMOX1* gene have also been identified, such as members of the heat-shock factor (HSF), nuclear factor-κB (NF-κB), and activator protein-1 (AP-1) families [58]. On the other hand, nemorosone was identified as a natural activator of the p300 histone acetyltransferase that enhanced histone acetylation in cells [59]. At the same time, p300-mediated NRF2 acetylation was shown to be essential for the maximal binding of NRF2 to specific ARE (antioxidant response element)-containing promoters [60]. Moreover, p300 was recently reported to compete with KEAP1 for the regulation of NRF2, enhancing the protein level of NRF2 and allowing NRF2 to translocate to the nucleus to upregulate the transcription of target genes [61]. This possible nemorosone-induced epigenetic regulation of the KEAP1–NRF2–HMOX1 axis could also explain the capacity of nemorosone to induce non-canonical ferroptosis through excessive activation of HMOX1. Consistently, we observed that nemorosone also induces the downregulation of *KEAP1*, *CUL3*, and *RBX1*, while it upregulates *SQSTM1* and *EIF2AK3* (PERK) (Figure 4B,D), all of which suggests the activation of the SQSTM1–KEAP1–NRF2–HMOX1 and PERK–NRF2–HMOX1 pathways as part of *HMOX1* overactivation-mediated cytotoxicity. It is important to highlight that the regulation of the expression at gene level of the KEAP1–CUL3–RBX1–NRF2 complex does not exclude the possibility of regulation at the protein level by a direct binding between nemorosone and KEAP1, as was reported in the aforementioned case of withaferin A [17]. All these factors need to be addressed in future experimental activities.

The time relation existing between the two ferroptosis mechanisms triggered by nemorosone is also noteworthy: the drop of GSH, resulting in a lipid peroxidation increase, appears from 2 h (an early event), while HMOX1 overexpression, with its consequent increase in the intracellular labile Fe^2+^ levels, only begins at 6 to 8 h (a later event). Moreover, before the execution of the later event, there is already cell death induction in some cells. However, the cell death level is accelerated and enhanced at the time points in which HMOX1 expression can be associated with labile Fe^2+^ and an additional lipid peroxidation increase. It is unclear whether this difference in cell death by early lipid peroxidation due to blockage of cystine import and by the later event represent two distinct responding populations, in which cell resistance to cell death during the early lipid peroxidation wave receives a second ferroptotic hit due to HMOX1 upregulation, hemin degradation, and the increase in the labile Fe^2+^ pool. Importantly, high sensitization values were achieved by combining nemorosone and the substrate of HMOX1 hemin, which confirms the cytotoxic role of nemorosone-induced HMOX1 activation and points out a possible therapeutic approach to be experimentally tested in in vivo experiments. The aforementioned results allow for the conclusion that nemorosone exerts an erastin-like ferroptosis (intrinsic ferroptosis) in fibrosarcoma cells that is characterized by the concurrence of both canonical (decreasing GSH levels) and non-canonical (increasing LIP through HMOX1 upregulation) mechanisms. This may confer more therapeutic efficacy to nemorosone by circumventing the resistance mechanisms of the tumor cells that bypass the System xc blockade or the depletion of GSH levels, an effect suggested by the persistence of the induction of cell death, unlike erastin, in the presence of β-ME (Figure 2D).

On the other hand, erastin-induced cell death and, in general, cysteine-deprivation-induced (CDI) ferroptosis are exerted by transient mitochondrial membrane potential (MMP) hyperpolarization, in such a way that low concentrations (10 μM) of the mitochondrial uncoupler CCCP can prevent (by the drop of MMP) CDI lipid ROS accumulation and protect against ferroptosis [23]. However, we confirmed that nemorosone acts as a mitochondrial uncoupler, dissipating (similar to CCCP) the transmembrane proton gradient prior to cell death execution. The possible involvement of mitochondrial uncoupling in ferroptosis induced by nemorosone was approached by using a high concentration of CCCP (50 μM) and methylnemorosone. While CCCP acted similarly to nemorosone regarding ferroptosis induction, methylnemorosone, which cannot exert mitochondrial uncoupling activity anymore, completely lost cytotoxicity. Altogether, this suggests that mitochondrial uncoupling is indeed required for nemorosone to trigger ferroptosis in fibrosarcoma cells. Furthermore, Figure S3 shows a possible link between HMOX1 over-activation and mitochondrial uncoupling: CCCP, a classic mitochondrial uncoupler, also increases Fe^2+^ levels upon *HMOX1* upregulation.

In addition, the obtained results at a high concentration of CCCP and the reported capacity to protect against erastin-induced ferroptosis at a low concentration [23] show a dual role as uncoupler compounds to induce ferroptosis or protect against it by varying the concentration. The protective mechanism could be an important approach to treat several ferroptosis-associated diseases such as ischemic organ injury, brain damage, and kidney failure [62,63], expanding the potential application of mitochondrial uncouplers.

To sum up, here, we connect, for the first time, mitochondrial uncoupling with ferroptotic cell death induction by the use of two closely related agents: proficient (nemorosone) and deficient (methylnemorosone). The cascade of cellular effects leading to ferroptosis induced by the mitochondrial uncoupler compounds in cancer cells is still an unexplored and emerging area of research and therapeutic opportunities.

## 5. Conclusions

Here, we show, for the first time, that nemorosone can induce intrinsic ferroptosis in fibrosarcoma and neuroblastoma cells by a double-edged targeting mechanism consisting of the drop of GSH as an early event and the increase in labile Fe^2+^ levels through the overexpression of HMOX1 as a later event. The work also expands the current knowledge about the role of mitochondria in ferroptosis by showing that compounds with an uncoupling action can trigger an erastin-like ferroptosis mechanism linked to MMP dissipation.

## Data Availability

The data presented in this study are available on request from the corresponding author.

## Supplementary Materials

The following supporting information can be downloaded at: https://www.mdpi.com/article/10.3390/cells12050735/s1, Figure S1: Nemorosone induces ferroptosis and mitochondrial uncoupling in neuroblastoma cells; Figure S2: Mitochondrial uncoupling triggered by nemorosone in fibrosarcoma cells; Figure S3: CCCP induces HMOX1 expression in HT1080 cells; Figure S4: RP-HPLC chromatogram of isolated (+)-nemorosone; Figure S5: ^1^H NMR spectrum of isolated (+)-nemorosone; Figure S6: ^13^C (APT) NMR spectrum of isolated (+)-nemorosone; Figure S7: ^1^H NMR spectrum of the crude mixture obtained after methylation of nemorosone; Figure S8: RP-HPLC chromatogram of obtained mixture of both O-methylated nemorosone isomers after chromatographic purification; Table S1: Selected ferroptosis-associated genes differentially expressed in HT1080 cells after exposition to nemorosone, CCCP and erastin in relation to the control (untreated cells); Table S2: *p*-values denoting the significance of enrichment of the differentially expressed pathways after treatment of HT1080 cells with nemorosone, CCCP and erastin.

## References

[1] De Oliveira C., Porto A., Bittrich V., Vencato I., Marsaioli A. (1996). Floral resins of *Clusia* spp.: Chemical composition and biological function. Tetrahedron Lett..

[2] Cuesta-Rubio O., Velez-Castro H., Frontana-Uribe B., Cárdenas J. (2001). Nemorosone, the major constituent of floral resins of *Clusia rosea*. Phytochemistry.

[3] Cuesta-Rubio O., Frontana-Uribe B., Ramírez-Apan T., Cárdenas J. (2002). Polyisoprenylated benzophenones in Cuban propolis; biological activity of nemorosone. Z. Naturforsch..

[4] Díaz-Carballo D., Malak S., Bardenheuer W., Freistuehler M., Reuscha H. (2008). Cytotoxic activity of nemorosone in neuroblastoma cells. J. Cell. Mol. Med..

[5] Holtrup F., Bauer A., Fellenberg K., Hilger R.A., Wink M., Hoheisel J. (2011). Microarray analysis of nemorosone-induced cytotoxic effects on pancreatic cancer cells reveals activation of the unfolded protein response (UPR). Br. J. Pharmacol..

[6] Pardo-Andreu G.L., Nuñez-Figueredo Y., Tudella V., Cuesta-Rubio O., Rodrigues F.P., Pestana C., Uyemura S., Leopoldino A., Alberici L., Curti C. (2011). The anti-cancer agent nemorosone is a new potent protonophoric mitochondrial uncoupler. Mitochondrion.

[7] Popolo A., Piccinelli A., Morello S., Sorrentino R., Cuesta-Rubio O., Rastrelli L., Aldo P. (2011). Cytotoxic activity of nemorosone in human MCF-7 breast cancer cells. Can. J. Physiol. Pharmacol..

[8] Camargo M., Oliveira M., Santoni M., Resende F., Oliveira-Höhne A., Espanha L., Nogueira C., Cuesta-Rubio O., Vilegas W., Varanda E. (2015). Effects of nemorosone, isolated from the plant *Clusia rosea*, on the cell cycle and gene expression in MCF-7 BUS breast cancer cell lines. Phytomedicine.

[9] Pardo-Andreu G.L., Reis F., Dalalio F., Nuñez Y., Cuesta-Rubio O., Uyemura S., Curti C., Alberici L. (2015). The cytotoxic effects of brown Cuban propolis depend on the nemorosone content and may be mediated by mitochondrial uncoupling. Chem. Biol. Interact..

[10] Frión-Herrera Y., Gabbia D., Díaz-García A., Cuesta-Rubio O., Carrara M. (2019). Chemosensitizing activity of Cuban propolis and nemorosone in doxorubicin resistant human colon carcinoma cells. Fitoterapia.

[11] Frión-Herrera Y., Gabbia D., Scaffidi M., Zagni L., Cuesta-Rubio O., Martin S.D., Carrara M. (2020). The Cuban propolis component nemorosone inhibits proliferation and metastatic properties of human colorectal cancer cells. Int. J. Mol. Sci..

[12] Camargo M., Varela S., Oliveira A.d., Resende F., Cuesta-Rubio O., Vilegas W., Varanda E.A. (2011). Assessment of estrogenic, mutagenic and antimutagenic activity of nemorosone. Rev. Bras. Farmacogn..

[13] Camargo M., Prieto A., Resende F., Boldrin P., Cardoso C., Fernández M., Molina-Molina J., Olea N., Vilegas W., Cuesta-Rubio O. (2013). Evaluation of estrogenic, antiestrogenic and genotoxic activity of nemorosone, the major compound found in brown cuban propolis. BMC Complement. Altern. Med..

[14] Díaz-Carballo D., Malak S., Freistühler M., Elmaagacli A., Bardenheuer W., Reusch H. (2008). Nemorosone blocks proliferation and induces apoptosis in leukemia cells. Int. J. Clin. Pharmacol. Ther..

[15] Lachaier E., Louandre C., Godin C., Saidak Z., Baert M., Diouf M., Chauffert B., Galmiche A. (2014). Sorafenib induces ferroptosis in human cancer cell lines originating from different solid tumors. Anticancer Res..

[16] Guo J., Xu B., Han Q., Zhou H., Xia Y., Gong C., Dai X., Li Z., Wu G. (2018). Ferroptosis: A novel anti-tumor action for cisplatin. Cancer Res. Treat..

[17] Hassannia B., Wiernicki B., Ingold I., Qu F., Herck S.V., Tyurina Y.Y., Bayır H., Abhari B.A., Angeli J.P.F., Choi S.M. (2018). Nano-targeted induction of dual ferroptotic mechanisms eradicates high-risk neuroblastoma. J. Clin. Investig..

[18] Ge C., Zhang S., Mu H., Zheng S., Tan Z., Huang X., Xu C., Zou J., Zhu Y., Feng D. (2022). Emerging mechanisms and disease implications of ferroptosis: Potential applications of natural products. Front. Cell Dev. Biol..

[19] Dixon S.J., Lemberg K.M., Lamprecht M.R., Skouta R., Zaitsev E.M., Gleason C.E., Patel D.N., Bauer A.J., Cantley A.M., Yang W.S. (2012). Ferroptosis: An iron-dependent form of nonapoptotic cell death. Cell.

[20] Wiernicki B., Dubois H., Tyurina Y.Y., Hassannia B., Bayir H., Kagan V.E., Vandenabeele P., Wullaert A., Berghe T.V. (2020). Excessive phospholipid peroxidation distinguishes ferroptosis from other cell death modes including pyroptosis. Cell Death Dis..

[21] Hassannia B., Coillie S.V., Berghe T.V. (2021). Ferroptosis: Biological rust of lipid membranes. Antioxid. Redox Signal..

[22] Hassannia B., Vandenabeele P., Berghe T.V. (2019). Targeting ferroptosis to iron out cancer. Cancer Cell.

[23] Gao M., Yi J., Zhu J., Minikes A.M., Monian P., Thompson C.B., Jiang X. (2019). Role of mitochondria in ferroptosis. Mol. Cell.

[24] Shrestha R., Johnson E., Byrne F.L. (2021). Exploring the therapeutic potential of mitochondrial uncouplers in cancer. Mol. Metab..

[25] Tsukano C., Siegel D.R., Danishefsky S.J. (2007). Differentiation of nonconventional “carbanions”-the total synthesis of nemorosone and clusianone. Angew. Chem. Int. Ed. Engl..

[26] Sparling B.A., Tucker J.K., Moebius D.C., Shair M.D. (2015). Total synthesis of (-)-nemorosone and (+)-secohyperforin. Org. Lett..

[27] Chena T.-C., Chuang J.-Y., Ko C.-Y., Kao T.-J., Yang P.-Y., Yu C.-H., Liu M.-S., Hu S.-L., Tsai Y.-T., Chan H. (2020). AR ubiquitination induced by the curcumin analog suppresses growth of temozolomide-resistant glioblastoma through disrupting GPX4-mediated redox homeostasis. Redox Biol..

[28] LingHu H.R., Luo H., Gang L. (2020). Bufalin induces glioma cell death by apoptosis or necroptosis. OncoTargets Ther..

[29] Grootjans S., Hassannia B., Delrue I., Goossens V., Wiernicki B., Dondelinger Y., Bertrand M.J.M., Krysko D.V., Vuylsteke M., Vandenabeele P. (2016). A real-time fluorometric method for the simultaneous detection of cell death type and rate. Nat. Protoc..

[30] Fernández-Acosta R., Iriarte-Mesa C., Alvarez-Alminaque D., Hassannia B., BartoszWiernicki, Díaz-García A.M., Vandenabeele P., Berghe T.V., Andreu G.L.P. (2022). Novel iron oxide nanoparticles induce ferroptosis in a panel of cancer cell lines. Molecules.

[31] Dykens J., Will Y. (2008). Drug-Induced Mitochondrial Dysfunction.

[32] Andrews S. A Quality Control Tool for High throughput Sequence Data. https://www.bioinformatics.babraham.ac.uk/projects/fastqc/.

[33] Bolger A.M., Lohse M., Usadel B. (2014). Trimmomatic: A flexible trimmer for Illumina sequence data. Bioinformatics.

[34] Dobin A., Davis C.A., Schlesinger F., Drenkow J., Zaleski C., Jha S., Batut P., Chaisson M., Gingeras T.R. (2013). STAR: Ultrafast universal RNA-seq aligner. Bioinformatics.

[35] Lawrence M., Huber W., Pagès H., Aboyoun P., Carlson M., Gentleman R., Morgan M.T., Carey V.J. (2013). Software for computing and annotating genomic ranges. PLoS Comput. Biol..

[36] Love M.I., Huber W., Anders S. (2014). Moderated estimation of fold change and dispersion for RNA-seq data with DESeq2. Genome Biol..

[37] Yant L.J., Ran Q., Rao L., Remmen H.V., Shibatani T., Belter J.G., Motta L., Richardson A., Prolla T.A. (2003). The selenoprotein GPX4 is essential for mouse development and protects from radiation and oxidative damage insults. Free Radic. Biol. Med..

[38] Lu S.C. (2009). Regulation of glutathione synthesis. Mol. Asp. Med..

[39] Yang W.S., SriRamaratnam R., Welsch M.E., Shimada K., Skouta R., Viswanathan V.S., Cheah J.H., Clemons P.A., Shamji A.F., Clish C.B. (2014). Regulation of ferroptotic cancer cell death by GPX4. Cell.

[40] Yang W.S., Kim K.J., Gaschler M.M., Patel M., Shchepinov M.S., Stockwell B.R. (2016). Peroxidation of polyunsaturated fatty acids by lipoxygenases drives ferroptosis. Proc. Natl. Acad. Sci. USA.

[41] Stockwell B.R., Angeli J.P.F., Bayir H., Bush A.I., Conrad M., Dixon S., Fulda S., Gascon S., Hatzios S.K., Kagan V. (2017). Ferroptosis: A regulated cell death nexus linking metabolism, redox biology, and disease. Cell.

[42] Dixon S.J., Patel D.N., Welsch M., Skouta R., Lee E.D., Hayano M., Thomas A.G., Gleason C.E., Tatonetti N.P., Slusher B.S. (2014). Pharmacological inhibition of cystine-glutamate exchange induces endoplasmic reticulum stress and ferroptosis. eLife.

[43] Sporn M.B., Liby K.T. (2012). NRF2 and cancer: The good, the bad and the importance of context. Nat. Rev. Cancer.

[44] Gozzelino R., Jeney V., Soares M.P. (2010). Mechanisms of Cell Protection By Heme Oxygenase-1. Annu. Rev. Pharmacol. Toxicol..

[45] Schulz S., Wong R.J., Vreman H.J., Stevenson D.K. (2012). Metalloporphyrins—An update. Front. Pharmacol..

[46] Stockwell B.R. (2022). Ferroptosis turns 10: Emerging mechanisms, physiological functions, and therapeutic applications. Cell.

[47] Zheng J., Sato M., Mishima E., Sato H., Proneth B., Conrad M. (2021). Sorafenib fails to trigger ferroptosis across a wide range of cancer cell lines. Cell Death Dis..

[48] Kato T., Shinoda J., Oka N., Miwa K., Nakayama N., Yano H., Maruyama T., Muragaki Y., Iwama T. (2008). Analysis of 11C-methionine uptake in low-grade gliomas and correlation with proliferative activity. AJNR Am. J. Neuroradiol..

[49] McBean G.J. (2012). The transsulfuration pathway: A source of cysteine for glutathione in astrocytes. Amino Acids.

[50] Pardo-Andreu G.L., Nuñez-Figueredo Y., Tudella V.G., Cuesta-Rubio O., Rodrigues F.P., Pestana C.R., Uyemura S.A., Leopoldino A.M., Alberici L.C., Curti C. (2011). The anti-cancer agent guttiferone-A permeabilizes mitochondrial membrane: Ensuing energetic and oxidative stress implications. Toxicol. Appl. Pharmacol..

[51] Fernández-Acosta R., Piñeros O., Pardo-Andreu G.L. (2019). The mitochondrial uncoupling as a promising pharmacological target against cancer. J. Pharm. Pharmacogn. Res..

[52] Shimada K., Hayano M., Pagano N.C., Stockwell B.R. (2016). Cell-line selectivity improves the predictive power of pharmacogenomic analyses and helps identify NADPH as biomarker for ferroptosis sensitivity. Cell Chem. Biol..

[53] Wei R., Zhao Y., Wang J., Yang X., Li S., Wang Y., Yang X., Fei J., Hao X., Zhao Y. (2021). Tagitinin C induces ferroptosis through PERK-Nrf2-HO-1 signaling pathway in colorectal cancer cells. Int. J. Biol. Sci..

[54] Kwon M.-Y., Park E., Lee S.-J., Chung S.W. (2015). Heme oxygenase-1 accelerates erastin-induced ferroptotic cell death. Oncotarget.

[55] Ngan K., Hoang L., Anstee J.E., Arnold J.N. (2021). The diverse roles of heme oxygenase-1 in tumor progression. Front. Immunol..

[56] Chiang S.-K., Chen S.-E., Chang L.-C. (2019). A dual role of heme oxygenase-1 in cancer cells. Int. J. Mol. Sci..

[57] Rouillard A.D., Gundersen G., Fernandez N.F., Wang Z., Monteiro C.D., McDermott M.G., Ma’ayan A. (2016). The harmonizome: A collection of processed datasets gathered to serve and mine knowledge about genes and proteins. Database.

[58] Alam J., Cook J.L. (2007). How many transcription factors does it take to turn on the heme oxygenase-1 gene?. Am. J. Respir. Cell Mol. Biol..

[59] Piaz F.D., Tosco A., Eletto D., Piccinelli A.L., Moltedo O., Franceschelli S., Sbardella G., Remondelli P., Rastrelli L., Vesci L. (2010). The identification of a novel natural activator of p300 histone acetyltranferase provides new insights into the modulation mechanism of this enzyme. ChemBioChem.

[60] Sun Z., Chin Y.E., Zhang D.D. (2009). Acetylation of Nrf2 by p300/CBP augments promoter-specific DNA binding of Nrf2 during the antioxidant response. Mol. Cell. Biol..

[61] Ganner A., Pfeiffer Z.-C., Wingendorf L., Kreis S., Klein M., Walz G., Neumann-Haefelin E. (2020). The acetyltransferase p300 regulates NRF2 stability and localization. Biochem. Biophys. Res. Commun..

[62] Friedmann J.P., Schneider M., Proneth B., Tyurina Y.Y., Tyurin V.A. (2014). Inactivation of the ferroptosis regulator Gpx4 triggers acute renal failure in mice. Nat. Cell Biol..

[63] Gao M., Monian P., Quadri N., Ramasamy R., Jiang X. (2015). Glutaminolysis and transferrin regulate ferroptosis. Mol. Cell.

