# Supplementary material for: Molecular Mechanisms of Nemorosone-Induced Ferroptosis in Cancer Cells
Source: Cells. 2023 Feb 24;12(5):735. doi: 10.3390/cells12050735 (PMC10000521; doi:10.3390/cells12050735)
Supplement: Supplementary file 1 [file cells-12-00735-s001.zip › cells-2121897-supplementary.pdf]

cells

MDPI

Supplementary Materials

Molecular Mechanisms of Nemorosone-induced Ferroptosis in Cancer Cells

Roberto Fernández-Acosta [1], Behrouz Hassannia [2,3,4], Jurgen Caroen [5], Bartosz Wiernicki [2,3], Daniel Alvarez-Alminaque [6], Bruno Verstraeten [2,3], Johan Van der Eycken [5], Peter Vandenabeele [2,3,7], Tom Vanden Berghe [2,3,4] and Gilberto L. Pardo-Andreu [6,*]

[1] Department of Pharmacy, Institute of Pharmacy and Food, University of Havana, 222 St. # 2317, La Coronela, La Lisa, Havana 13600, Cuba
[2] Cell Death and Inflammation Unit, VIB Center for Inflammation Research (IRC), 9052 Ghent, Belgium
[3] Department of Biomedical Molecular Biology (DBMB), Ghent University, 9052 Ghent, Belgium
[4] Laboratory of Pathophysiology, Department of Biomedical Sciences, University of Antwerp, 2000 Antwerp, Belgium
[5] Laboratory for Organic and Bio-Organic Synthesis, Department of Organic and Macromolecular Chemistry, Ghent University, 9000 Ghent, Belgium
[6] Center for Research and Biological Evaluations, Institute of Pharmacy and Food, University of Havana, 222 St. # 2317, La Coronela, La Lisa, Havana 13600, Cuba
[7] Methusalem Program, Ghent University, 9052 Ghent, Belgium
* Correspondence: gpardo@ifal.uh.cu

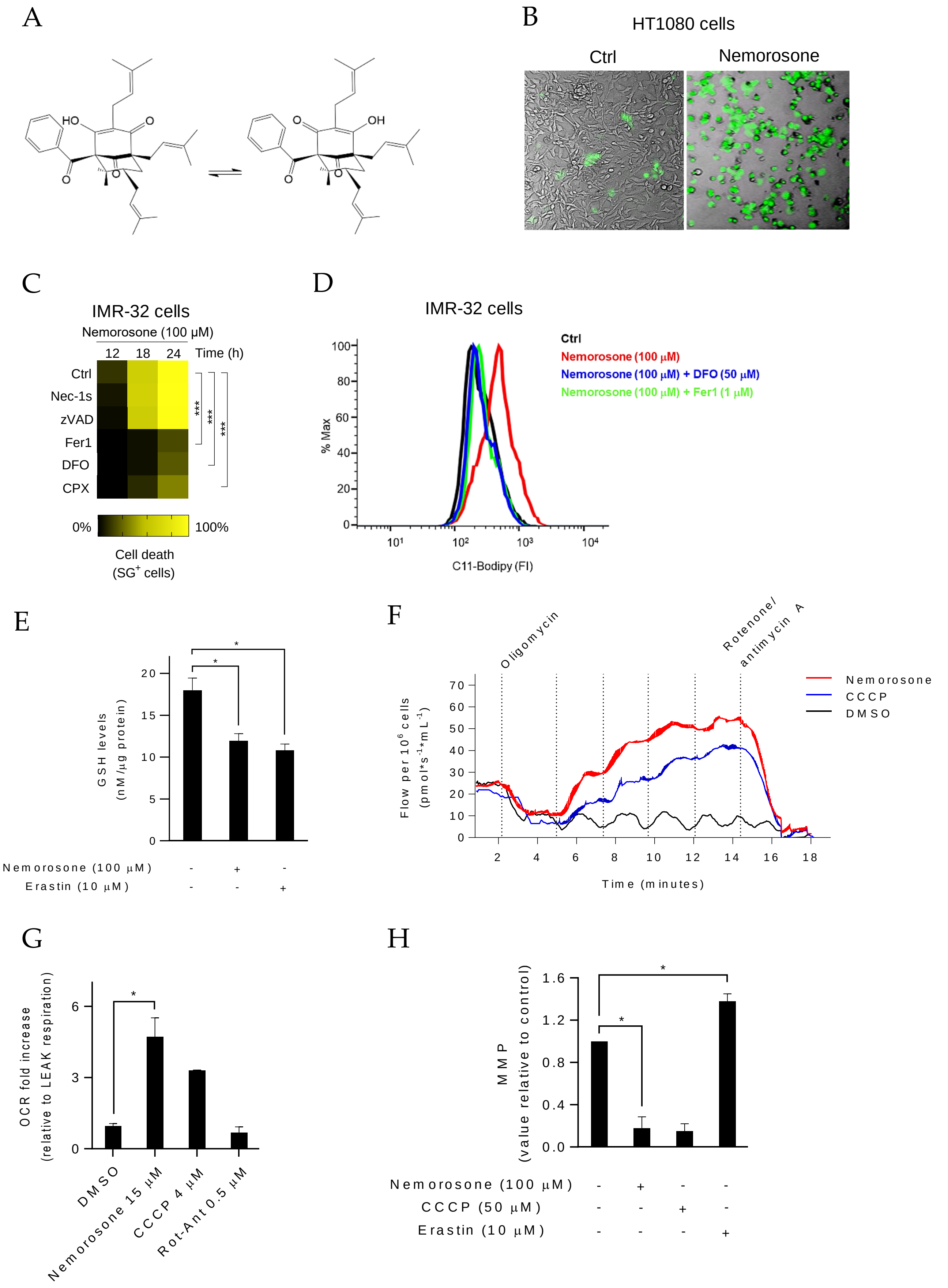

Figure S1. Nemorosone induces ferroptosis and mitochondrial uncoupling in neuroblastoma cells. (**A**) Nemorosone's structure (mixture of tautomers): left: (1*S*,5*R*,7*R*)-5-benzoyl-4-hydroxy-6,6-

dimethyl-1,3,7-tris(3-methylbut-2-en-1-yl)bicyclo[3.3.1]non-3-ene-2,9-dione; right: (1*S*,5*S*,7*R*)-1-benzoyl-4-hydroxy-8,8-dimethyl-3,5,7-tris(3-methylbut-2-en-1-yl)bicyclo[3.3.1]non-3-ene-2,9-dione. (**B**) Snapshots from live cell imaging of untreated (Ctrl) and nemorosone-treated HT1080 cells (12 h, 100 µM). Green fluorescent staining represents SytoxGreen dye. (**C**) Heatmap representing cell death sensitivity of neuroblastoma (IMR-32) cells after exposure to 100 µM of nemorosone, in the absence or presence of different inhibitors: the necroptosis inhibitor: necrostatin-1 (Nec-1s, 10 µM), the pan-caspase inhibitor Z-VAD-FMK (10 µM), and the ferroptosis inhibitors ferrostatin-1 (Fer1, 1 µM), deferoxamine (DFO, 50 µM), and ciclopirox olamine (CPX, 5 µM). (**D**) Flow cytometry analysis (histogram) of the lipid peroxidation sensor (C11-BODIPY-581/591 dye) on live-gated IMR-32 cells (DRAQ7-negative cells) after treatment with nemorosone (2 h, 100 µM) and its combination with the ferroptosis inhibitors DFO (50 µM) and Fer1 (1 µM). FI is the fluorescent intensity. Traces are representative of two independent experiments. (**E**) GSH levels in IMR-32 cells after treatment with nemorosone (2 h, 100 µM) or erastin (4 h, 10 µM). (**F**) Phosphorylation control protocol performed by high-resolution respirometry in intact IMR-32 cells. After the addition of oligomycin (1.5 µM), cells were titrated with nemorosone and CCCP in steps of 5 µM and 1 µM additions respectively, totalizing final concentrations of 20 µM (nemorosone) and 4 µM (CCCP) in the Oxygraph-2k chambers (2 mL). DMSO was added similar to nemorosone. The O_2 flow (pmol*s^{-1}*mL^{-1}) was inhibited to a constant level after the addition of rotenone-antimycin A mix (0.5 µM). Every addition is represented by vertical dot lines. (**G**) Oxygen consumption rate (OCR) fold increase relative to LEAK state (reached after the inhibition of F_1F_O ATP synthase by oligomycin) in IMR-32 cells exerted by the separated addition of DMSO (15 µM), nemorosone (15 µM), CCCP (4 µM) and rotenone-antimycin A mix (Rot-Ant, 0.5 µM). (**H**) Flow cytometry analysis of the mitochondrial membrane potential sensor (TMRE) on live-gated IMR-32 cells (SytoxBlue-negative cells) after treatment with nemorosone (1 h, 100 µM), CCCP (1 h, 50 µM), or erastin (3 h, 10 µM). Values are expressed as the fold increase of each signal relative to the median fluorescence intensity of the control. The combined results of 2 independent experiments are shown for (**C**) and (**E**)-(**H**). All quantitative data are presented as mean ± SD. *p < 0.05, ***p < 0.001 by unpaired Student's t-test.

.

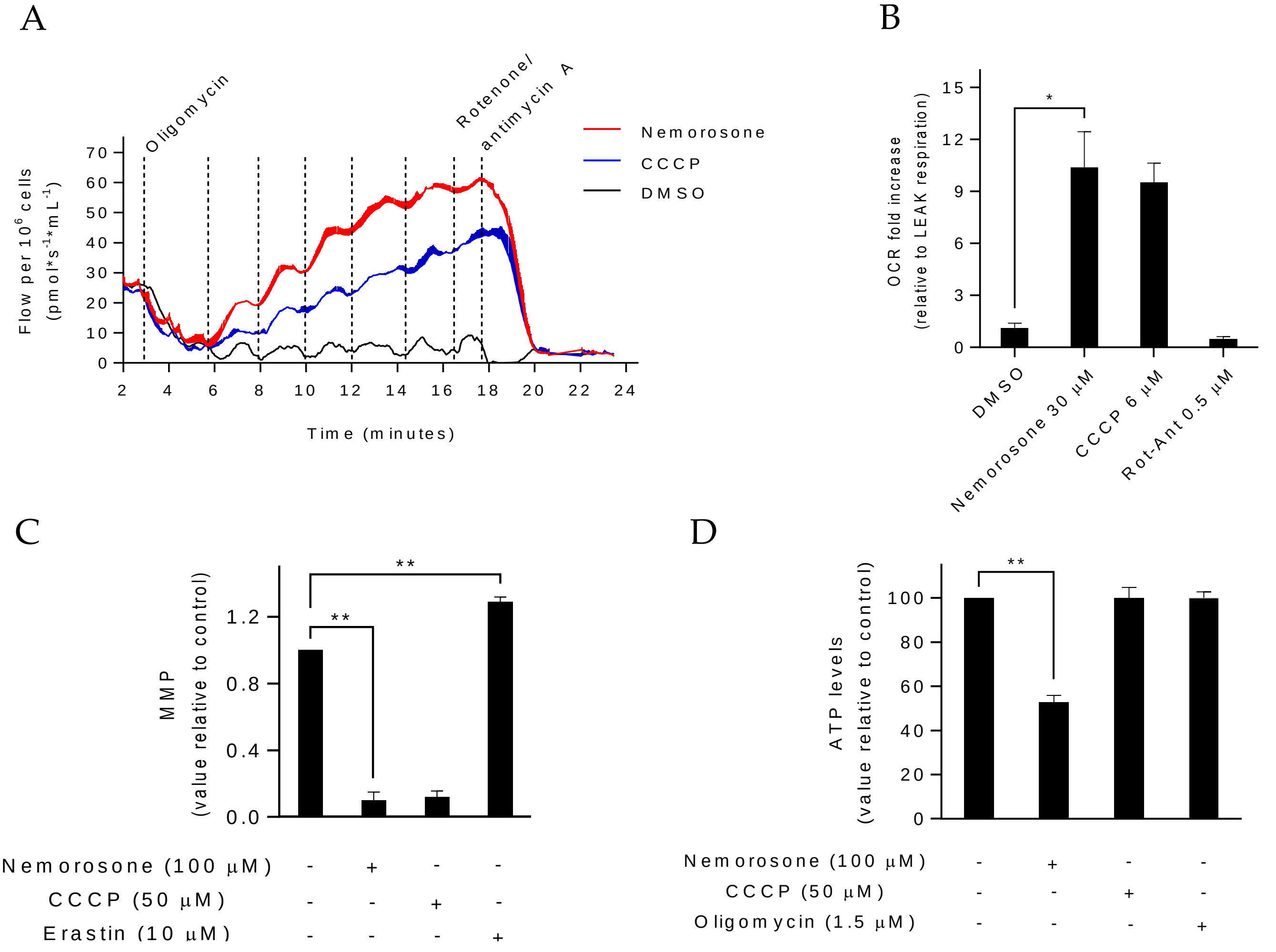

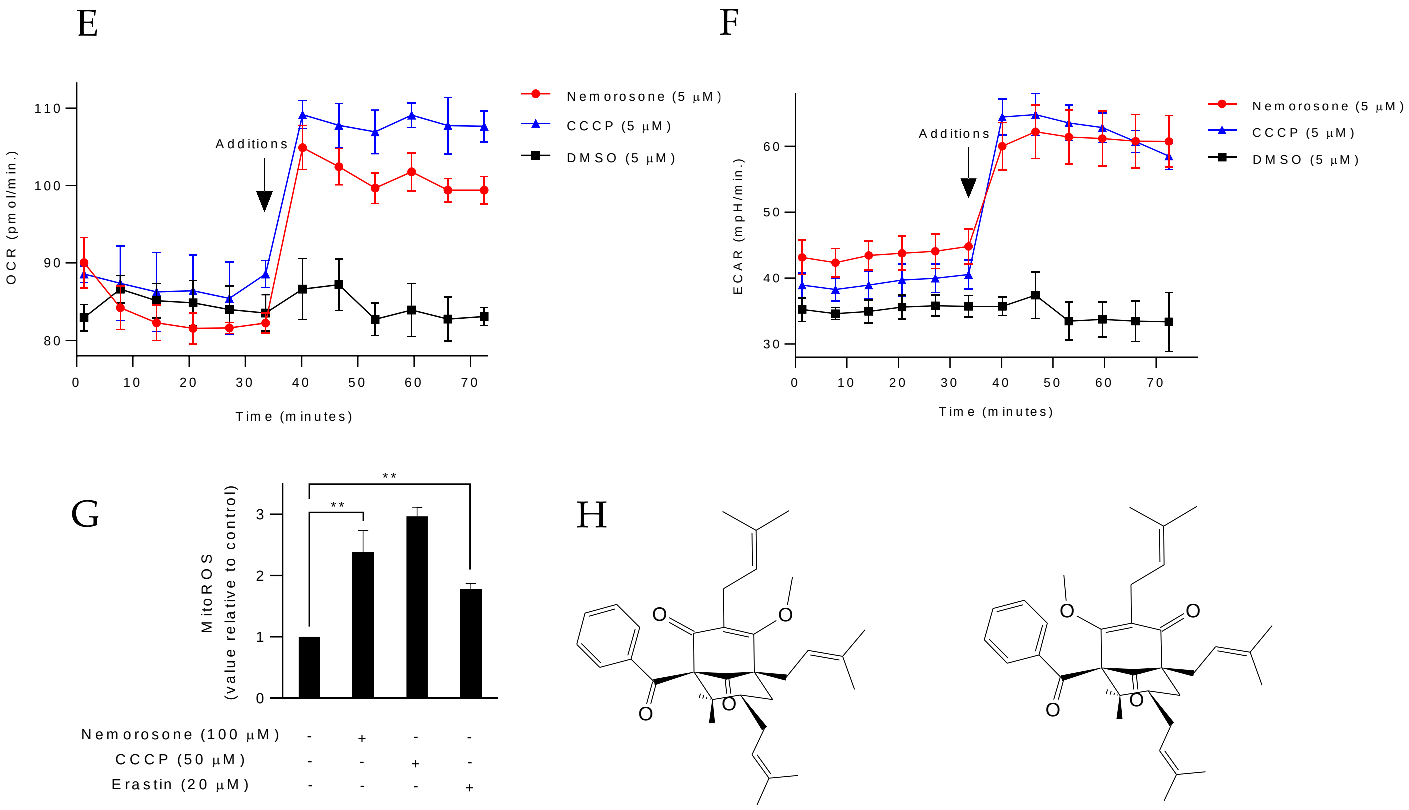

Figure S2. Mitochondrial uncoupling triggered by nemorosone in fibrosarcoma cells. (**A**) Phosphorylation control protocol performed by high-resolution respirometry in intact HT1080 cells. Experimental conditions similar to those described in Supplementary Fig. 1f. Nemorosone and CCCP were added in steps of 5 µM and 1 µM respectively, totalizing final concentrations of 30 µM (nemorosone) and 6 µM (CCCP). (**B**) Oxygen consumption rate (OCR) fold increase relative to LEAK state (reached after the inhibition of F_1F_O ATP synthase by oligomycin) in HT1080 cells exerted by the separated addition of DMSO (30 µM), nemorosone (30 µM), CCCP (6 µM) and rotenone-antimycin A mix (Rot-Ant, 0.5 µM). (**C**) Flow cytometry analysis of the mitochondrial membrane potential sensor (TMRE) on live-gated HT1080 cells (SytoxBlue-negative cells) after treatment with nemorosone (1 h, 100 µM), CCCP (1 h, 50 µM), or erastin (3 h, 10 µM). Values are expressed as the fold increase of each signal relative to the median fluorescence intensity of the control. (**D**) ATP levels in HT1080 cells after 6 h of treatment in the absence (control) or presence of nemorosone (100 µM), CCCP (50 µM) or oligomycin (1.5 µM). (**E**), (**F**) OCR and extracellular acidification rate (ECAR), respectively, in HT1080 cells treated with nemorosone (5 µM) and CCCP (5 µM) as measured using Seahorse XF flux analyzer. Compounds additions are indicated by arrows. OCR and ECAR values are representative rates of n = 6-8 wells/treatment/experiment from one experiment. (**G**) Flow cytometry analysis of the mitochondrial superoxide production (mitoROS) sensor MitoSox Red on live-gated HT1080 cells (SytoxBlue-negative cells) after treatment with nemorosone (2 h, 100 µM), CCCP (2 h, 50 µM) or erastin (2 h, 20 µM). Values are expressed as the fold increase of each signal relative to the median fluorescence intensity of the control. (**H**) Methylnemorosone's structure, a mixture of 2 isomers: left: (1*S*,5*S*,7*R*)-1-benzoyl-4-methoxy-8,8-dimethyl-3,5,7-tris(3-methylbut-2-en-1-yl)bicyclo[3.3.1]non-3-ene-2,9-dione; right: (1*S*,5*R*,7*R*)-5-benzoyl-4-methoxy-6,6-dimethyl-1,3,7-tris(3-methylbut-2-en-1-yl)bicyclo[3.3.1]non-3-ene-2,9-dione. The combined results of 3 independent experiments are shown for (**A**)-(**D**) and (**G**). All quantitative data are presented as mean ± SD. *$p < 0.05$, **$p < 0.01$ by unpaired Student's t-test.

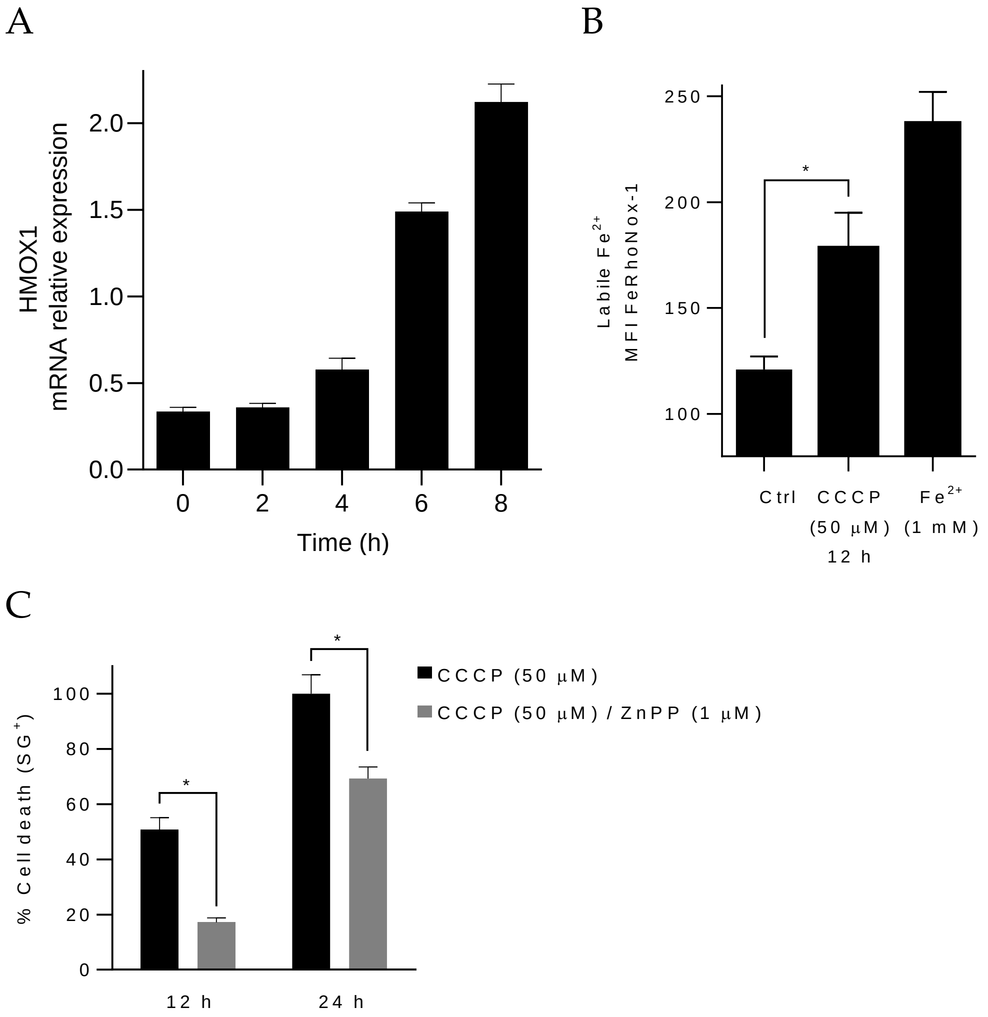

Figure S3. CCCP induces HMOX1 expression in HT1080 cells. (**A**) Relative *HMOX1* mRNA expression in HT1080 cells after CCCP (50 µM) treatment at different time points. (**B**) Cellular levels of labile Fe^{2+}, determined by flow cytometry by using FeRhoNox-1 dye, in response to CCCP (12 h, 50 µM). (MFI is the median fluorescence intensity.) Ferrous ammonium sulfate was used as a control (2 h ,1 mM). (**C**) Percentage of cell death in function of time induced by CCCP (50 µM) in the presence/absence of ZnPP (1 µM), assessed by using SytoxGreen dye. The combined results of 2 independent experiments are shown for (**B**) and (**C**). All quantitative data are presented as mean ± SD. *$p < 0.05$ by unpaired Student's t-test.

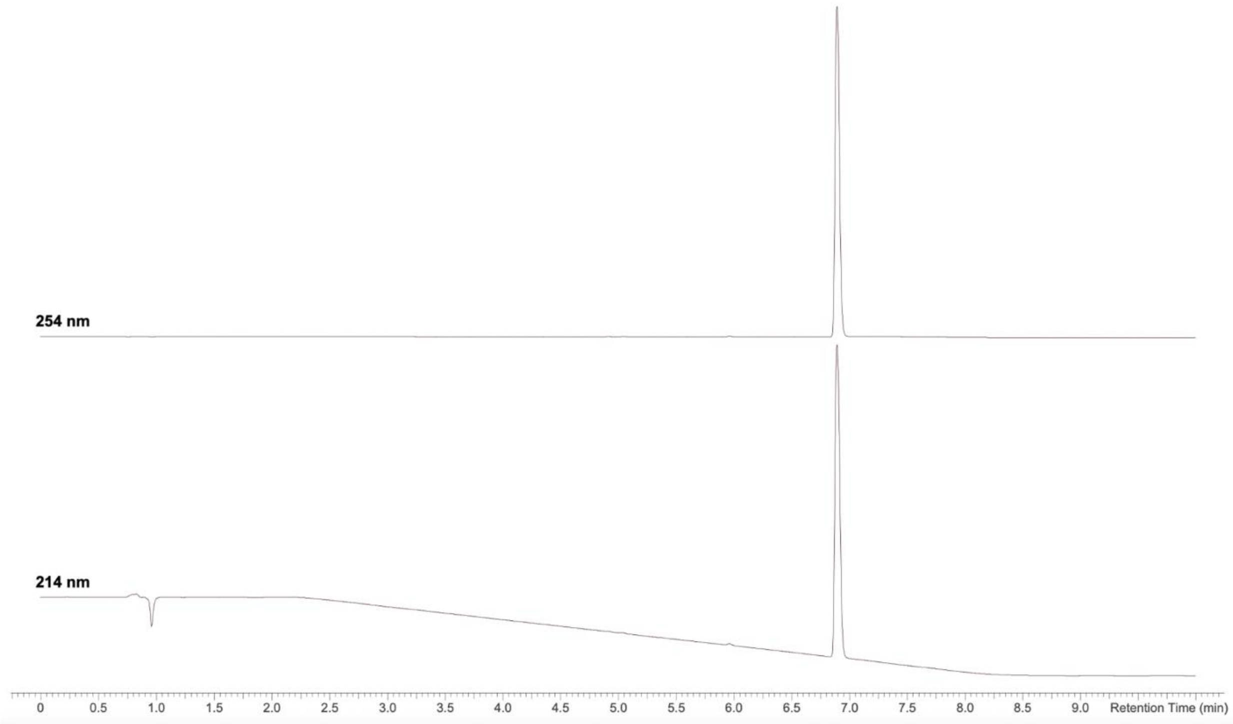

Figure S4. RP-HPLC chromatogram of isolated (+)-nemorosone, retention time: 6.9 min. Purity 99.8% (integration of peaks at 214 nm). Eluting conditions: eluent A/eluent B (50/50) during 30 s, followed by gradient elution (A/B from 50/50 to 0/100) over 6 minutes (eluent A: 0.1% HCOOH in water; eluent B: acetonitrile) on a Phenomenex Luna C18 (2), 5 µm 250 mm x 4.60 mm column.

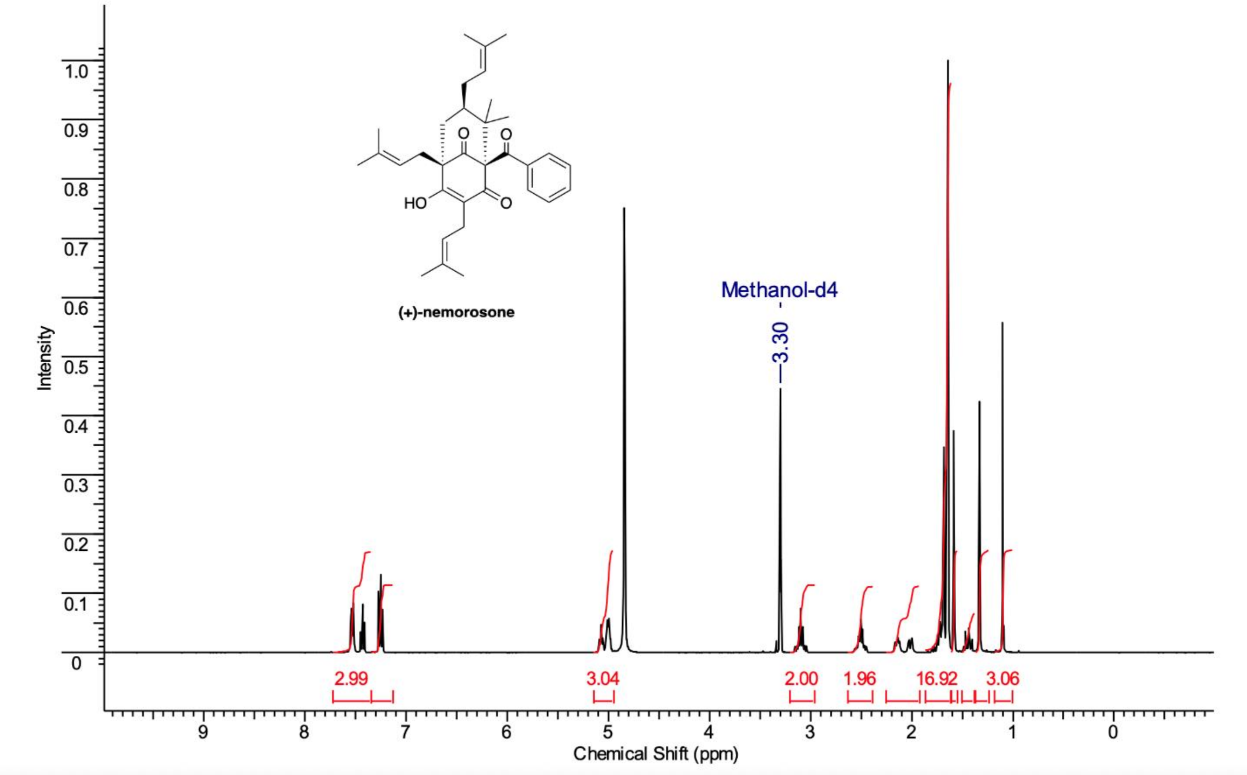

Figure S5. ^{1}H NMR spectrum of isolated (+)-nemorosone (400MHz, CD_3OD).

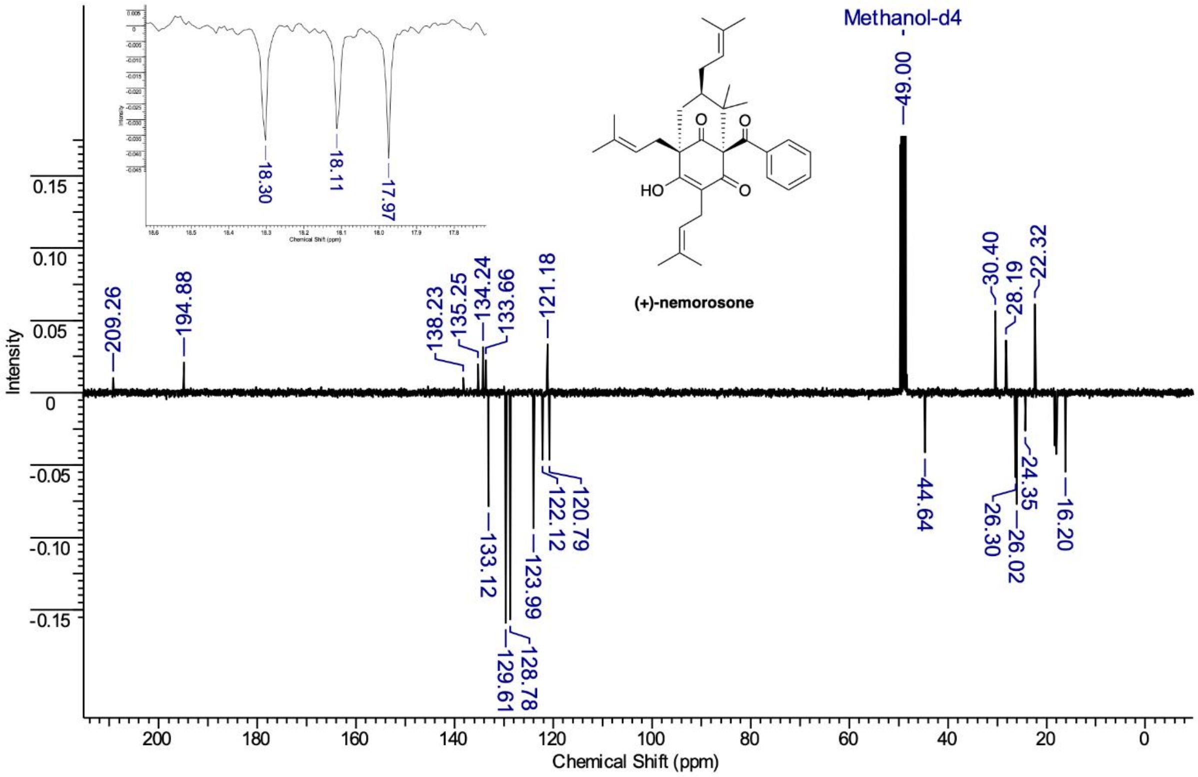

Figure S6. ^{13}C (APT) NMR spectrum of isolated (+)-nemorosone (100MHz, CD_3OD).

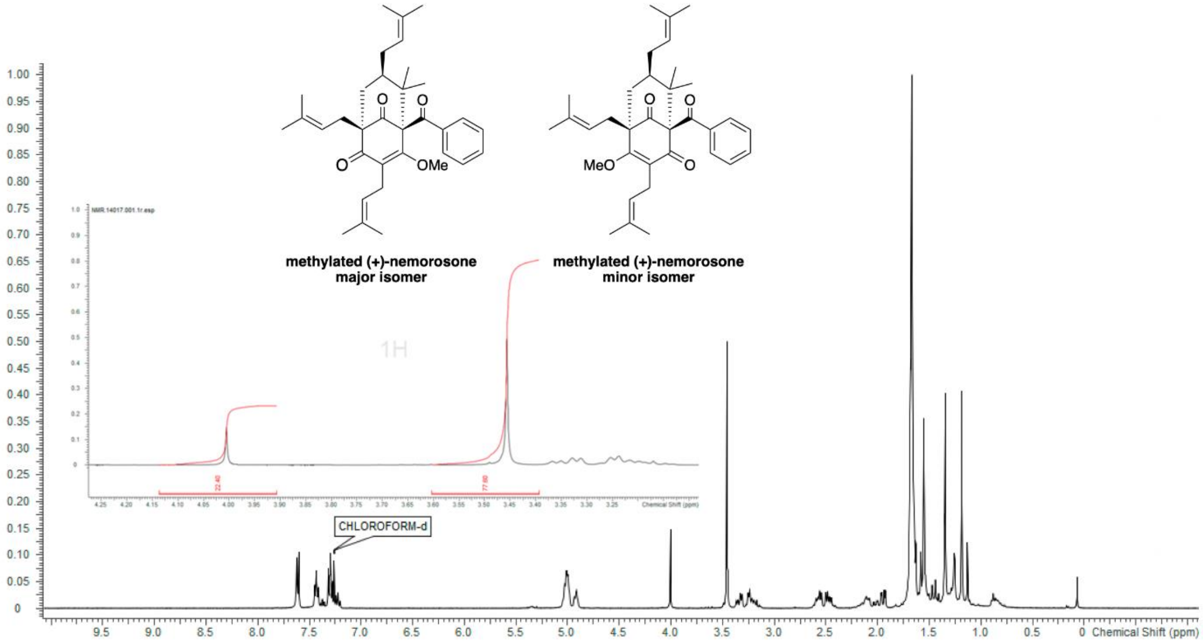

Figure S7. ^{1}H NMR spectrum (400MHz, $CDCl_3$) of the crude mixture obtained after methylation of nemorosone (before chromatographic purification). Inserted detail: integration of diagnostic singlet signals corresponding to the vinylogous methyl ester of both isomers (ratio: 78/22).

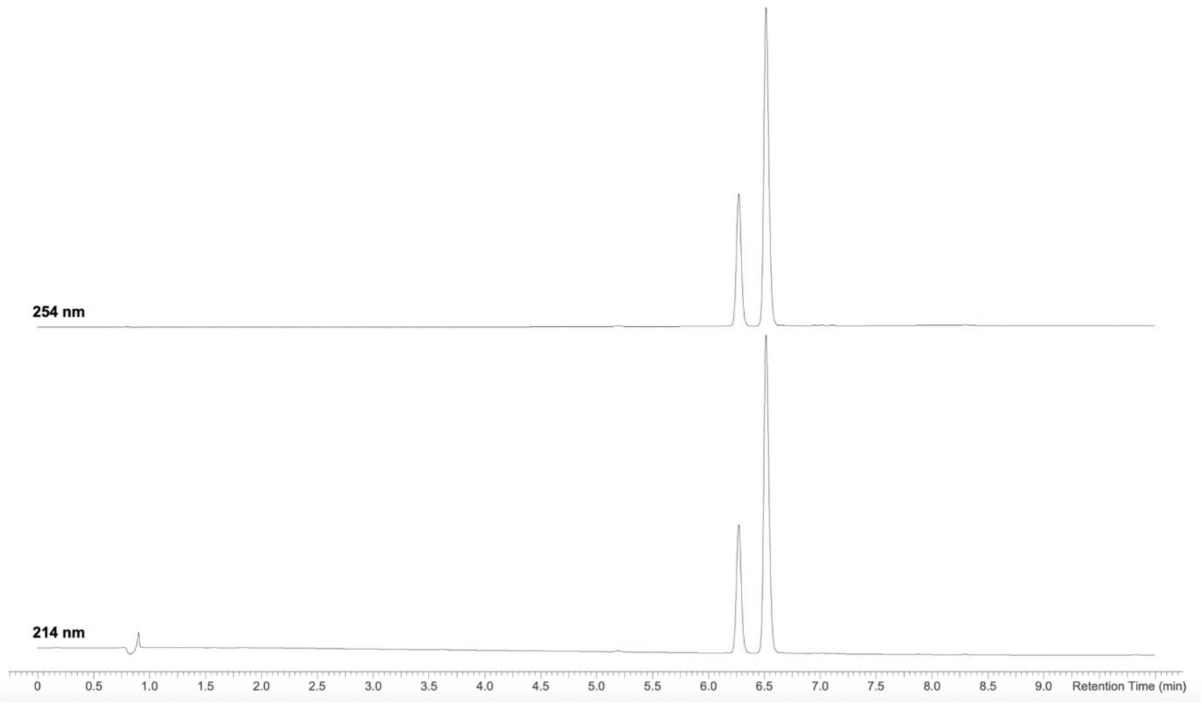

Figure S8. RP-HPLC chromatogram of obtained mixture of both *O*-methylated nemorosone isomers after chromatographic purification. Retention times: 6.3 min and 6.5 min (purity 99.3%, ratio: 27.6/72.4, integration of peaks at 214 nm). Eluting conditions: eluent A/eluent B (100/0) during 30 s, followed by gradient elution (A/B from 100/0 to 0/100) over 6 minutes (eluent A: 5 mM NH_4OAc in water; eluent B: acetonitrile) on a Phenomenex Luna C18 (2), 5 µm 250 mm x 4.60 mm column. This mixture was used for biological testing.

Table S1. Selected ferroptosis-associated genes differentially expressed in HT1080 cells after exposition to nemorosone (100 μM, 8 h), CCCP (50 μM, 8 h) and erastin (20 μM, 8 h) in relation to the control (untreated cells). LFC: log_2fold-change, metric indicating how much the expression of a gene changes compared to the control condition; positive values indicate higher expression than the control, while negative values indicate lower expression than the control. When the gene was not differentially expressed by the treatment in question, a dash appears in place of the value. HUGO Gene Nomenclature Committee (HGNC) symbols were used to designate genes. In the case of *SLC7A11* and *ATF3*, the value obtained at 2 h of treatment is shown on the left, and the value obtained at 8 h of treatment is shown on the right.

	Gen (HGNC)	LFC (nemorosone)	LFC (CCCP)	LFC (erastin)
**NRF2-mediated oxidative stress response**	*SQSTM1*	1.41	0.46	0.42
	KEAP1	-0.57	-	-
	CUL3	-0.70	-0.39	-
	RBX1	-0.44	-0.29	-
	EIF2AK3	2.68	1.21	-
	NRF2	1.11	0.69	-
	HMOX1	6.55	4.04	1.79
	FTH1	1.54	0.53	0.32
	FTL	0.73	0.38	-
	TFRC	0.44	0.69	-0.29
	NCOA4	0.19	-	-
	GCLC	1.10	0.74	-
	GCLM	2.00	1.40	0.64
	CDKN1A	0.61	-	-
**p53 signaling**	*TP53*	-	0.57	-
	SOCS1	2.06	-	-
	SLC7A11	-0.31 0.58	- 0.90	-
	SAT1	3.50	1.74	-
	ATF3	3.17 4.48	3.03 2.31	-
	SERPINE1	2.24	1.30	-
	GADD45A	3.63	1.67	-
	GADD45B	2.22	1.01	-
	MDM2	0.19	0.35	-
	MDM4	1.19	-	-
**HSP-mediated response**	*ATF4*	0.56	0.37	-
	HSPA5	3.58	0.44	0.79
	HSPA6	5.93	3.33	-
	HSPA1A	4.52	2.49	1.08
	HSPA1B	4.28	1.97	-
	HSP90AA1	1.28	0.48	-
	HSP90AB1	0.54	0.29	-
	LAMP2	-	0.17	-
	HSPA8	1.22	-	-

	Gen (HGNC)	LFC (nemorosone)	LFC (CCCP)	LFC (erastin)
FSP1	*AIFM2 (FSP1)*	1.16	0.52	-
**Unfolded protein response**	*ERN1* (IRE1α)	2.69	1.78	-
	ATF6	0.27	0.68	-
	DDIT3	5.40	3.55	-
**Apoptosis signaling**	*BCL2L11*	-0.38	-	-
	CASP2	-1.48	-1.22	-
	CASP3	1.02	0.48	-
	CASP6	-0.93	-0.95	
	CASP7	-1.02	-	-
	CASP9	-0.79	-	-
	APAF1	-1.21	-	-
	CFLAR	1.47	0.62	-
	XIAP	0.64	0.32	-
	MCL1	0.57	0.23	-0.20
	BCL2L11	-0.38	-	-

Table S2. p values denoting the significance of enrichment of the differentially expressed pathways after treatment of HT1080 cells with nemorosone (100 μM, 8 h), CCCP (50 μM, 8 h) and erastin (20 μM, 8 h). When the pathway was not differentially expressed by the treatment in question, a dash appears in place of the value.

Differentially expressed pathway	p value (nemorosone)	p value (CCCP)	p value (erastin)
Ferroptosis signaling pathway	8×10^{-8}	2×10^{-7}	4×10^{-4}
Autophagy	4×10^{-10}	4×10^{-14}	1×10^{-2}
Necroptosis signaling pathway	1×10^{-2}	3×10^{-3}	-
Apoptosis signaling	6×10^{-5}	4×10^{-3}	-
Endoplasmic reticulum stress pathway	6×10^{-9}	4×10^{-4}	4×10^{-4}
Unfolded protein response	1×10^{-12}	5×10^{-9}	4×10^{-8}
NRF2-mediated oxidative stress response	3×10^{-8}	4×10^{-10}	4×10^{-3}
PI3K/AKT signaling	6×10^{-7}	2×10^{-6}	8×10^{-3}
p53 signaling	2×10^{-11}	8×10^{-9}	-
mTOR signaling	2×10^{-12}	1×10^{-7}	-
HIF1a signaling	9×10^{-5}	2×10^{-8}	2×10^{-4}
Oxidative phosphorylation	4×10^{-2}	3×10^{-5}	3×10^{-3}
